# An inducible mouse model of osteogenesis imperfecta type V reveals aberrant osteogenesis caused by *Ifitm5* c.-14C>T mutation

**DOI:** 10.1093/jbmr/zjaf022

**Published:** 2025-02-05

**Authors:** Zhijia Tan, Hiu Tung Shek, Zeluan Li, Linjian Xia, Yanni He, Peikai Chen, Janus Siu Him Wong, Bo Gao, Danny Chan, Michael Kai Tsun To

**Affiliations:** Department of Orthopaedics and Traumatology, The University of Hong Kong-Shenzhen Hospital, Shenzhen 518053, China; Shenzhen Clinical Research Centre for Rare Diseases, The University of Hong Kong-Shenzhen Hospital, Shenzhen 518053, China; Department of Orthopaedics and Traumatology, Li Ka Shing Faculty of Medicine, The University of Hong Kong, Hong Kong, China; Shenzhen Clinical Research Centre for Rare Diseases, The University of Hong Kong-Shenzhen Hospital, Shenzhen 518053, China; School of Biomedical Sciences, Li Ka Shing Faculty of Medicine, The University of Hong Kong, Hong Kong, China; Shenzhen Clinical Research Centre for Rare Diseases, The University of Hong Kong-Shenzhen Hospital, Shenzhen 518053, China; Department of Orthopaedics and Traumatology, Li Ka Shing Faculty of Medicine, The University of Hong Kong, Hong Kong, China; Clinical, Translational and Basic Research Laboratory, The University of Hong Kong-Shenzhen Hospital, Shenzhen 518053, China; Department of Orthopaedics and Traumatology, The University of Hong Kong-Shenzhen Hospital, Shenzhen 518053, China; Shenzhen Clinical Research Centre for Rare Diseases, The University of Hong Kong-Shenzhen Hospital, Shenzhen 518053, China; Department of Orthopaedics and Traumatology, The University of Hong Kong-Shenzhen Hospital, Shenzhen 518053, China; Shenzhen Clinical Research Centre for Rare Diseases, The University of Hong Kong-Shenzhen Hospital, Shenzhen 518053, China; School of Biomedical Sciences, Li Ka Shing Faculty of Medicine, The University of Hong Kong, Hong Kong, China; Department of Orthopaedics and Traumatology, The University of Hong Kong-Shenzhen Hospital, Shenzhen 518053, China; Shenzhen Clinical Research Centre for Rare Diseases, The University of Hong Kong-Shenzhen Hospital, Shenzhen 518053, China; Department of Orthopaedics and Traumatology, Li Ka Shing Faculty of Medicine, The University of Hong Kong, Hong Kong, China; Department of Orthopaedics and Traumatology, The University of Hong Kong-Shenzhen Hospital, Shenzhen 518053, China; School of Biomedical Sciences, Faculty of Medicine, The Chinese University of Hong Kong, Hong Kong, China; Department of Orthopaedics and Traumatology, The University of Hong Kong-Shenzhen Hospital, Shenzhen 518053, China; School of Biomedical Sciences, Li Ka Shing Faculty of Medicine, The University of Hong Kong, Hong Kong, China; Department of Orthopaedics and Traumatology, The University of Hong Kong-Shenzhen Hospital, Shenzhen 518053, China; Shenzhen Clinical Research Centre for Rare Diseases, The University of Hong Kong-Shenzhen Hospital, Shenzhen 518053, China; Department of Orthopaedics and Traumatology, Li Ka Shing Faculty of Medicine, The University of Hong Kong, Hong Kong, China

**Keywords:** type V osteogenesis imperfecta, IFITM5, inducible mouse model, skeletal progenitor, cell fate

## Abstract

Osteogenesis imperfecta (OI) type V is typically characterized by radial head dislocation, calcification of interosseous membrane, and hyperplastic callus. It is caused by the c.-14C>T mutation in the 5′ UTR of *IFITM5* gene, adding 5 amino acids (MALEP) to the N-terminal of IFITM5 protein. Previous studies have suggested a neomorphic function of the MALEP-IFITM5 protein. However, the underlying mechanisms remain unclear due to embryonic lethality in previous mouse models. Therefore, we developed an inducible mouse model (*Ifitm5^flox c.-14C>T^*) that could be induced by Cre expressed at different developmental stages to explore the pathogenic effects of the neomorphic MALEP-IFITM5. The mutant *Ifitm5* allele could be regulated by the endogenous regulatory elements after Cre recombination, maintaining its spatiotemporal expression pattern and physiological level. Specifically, *Prx1-Cre; Ifitm5^flox c.-14C>T^* mutant mice were born with fractures in all limbs, showing impaired ossification and enhanced chondrogenesis associated with increased SOX9 abundance. Analyses of single-cell RNA sequencing data revealed arrested osteogenesis in *Prx1-Cre; Ifitm5^flox c.-14C>T^* mouse. A major population of cells expressing both osteogenic and chondrogenic signature genes was identified in the mutant mouse. Reduced expression of SP7 and SOST in the cortical regions of mutant mice confirmed delayed osteocyte maturation and compromised osteogenesis. Elevated bone marrow adipocytes were found in the adult mutant mice. Ectopic chondrogenesis and SOX9 expression were also observed in the perichondrium regions of *Col1a1-Cre; Ifitm5^flox c.-14C>T^* and *Ocn-Cre; Ifitm5^flox c.-14C>T^* mutant mice. The inducible *Ifitm5^flox c.-14C>T^* mouse model and integrated single-cell transcriptomic analyses elucidated that ectopic expression of SOX9 and disrupted homeostatic balance among osteogenesis, chondrogenesis, and adipogenesis may contribute to the pathogenesis caused by MALEP-IFITM5, helping to gain deeper insights into the molecular mechanisms of type V OI.

## Introduction

Bone forms in two basic but distinct ways: intramembranous ossification and endochondral ossification, both of which begin with the mesenchymal condensation. In the process of endochondral bone formation, the osteochondral progenitors first commit to chondrocytes, and undergo a series of coordinated differentiation processes. During chondrocyte hypertrophy, progenitors in the adjacent perichondrium differentiate into osteoblasts and form mineralized cortical bone.^1^ At the osteochondral junction, hypertrophic chondrocytes direct the invasion of blood vessels, bringing in the precursors of osteoclasts and osteoblasts to remodel the cartilage and form primary spongiosa.^2^ During bone growth, homeostasis is mainly maintained by the balance between bone formation and bone resorption. OI is an inherited bone disorder characterized by low bone mass, recurrent fractures, and long bone deformities.^3^^,^^4^ Genetic analyses have identified more than 20 OI causative genes involved in type I collagen synthesis (*COL1A1* and *COL1A2*), posttranslational modification and processing (*CRTAP*, *P3H1*, and *PPIB*), bone mineralization (*IFITM5*), and osteoblast differentiation (*SP7*, *CREB3L1*, and *WNT1*).^4–7^

Type V OI (OMIM 610967) was first described by Glorieux et al.^8^ in 2000 with distinct clinical features including hyperplastic callus after fracture, interosseous membrane calcification, and radial head dislocation. It is an autosomal dominant form of OI presenting moderate to severe manifestations. Genetic studies have confirmed that type V OI was caused by a point mutation in the 5′ UTR of *IFITM5* gene (c.-14C>T), resulting in the addition of 5 amino acids (Met-Ala-Leu-Glu-Pro, MALEP) at its N-terminus.^9–12^ IFITM5 (BRIL, Interferon Induced Transmembrane Protein 5) is a palmitoylated type II transmembrane protein and specifically expressed in mature osteoblasts.^13–15^  *In vitro* differentiation assays suggest that IFITM5 promotes osteoblast maturation and matrix mineralization.^13^ However, *Ifitm5*-deficient mice (*Ifitm5^-/-^*) did not exhibit significant skeletal abnormalities. Transgenic mice (*Ifitm5^c.-14C>T^* driven by *Col1a1* promoter) and CRISPR-Cas9 engineered knock-in mice (*Ifitm5^c.-14C>T^*) exhibit severe skeletal defects and perinatal lethality. Osteogenesis was attenuated in these mutant mice,^16–18^ suggesting the neomorphic function of the MALEP-IFITM5 protein. The embryonic lethality greatly hinders the exploration of the underlying mechanisms in type V OI. Researchers generated a conditional mouse model (Rosa26-*Ifitm5^c.-14C>T^*) recently and found the induction of mutant IFITM5 in the osteo-chondroprogenitors or chondrogenic lineages resulted in cartilage overgrowth and joint deformity, which was partially driven by elevated ERK-SOX9 activities.^19^ However, IFITM5 (BRIL) is a bone restricted protein.^13^ The Rosa26-*Ifitm5^c.-14C>T^* model may change the endogenous expression pattern of IFITM5. Therefore, developing a more optimal animal model would help to gain a deeper insight into the pathogenesis of type V OI.

In this study, we generated a mouse line (*Ifitm5^flox c.-14C>T^*) with c.-14C>T mutation in the *Ifitm5* locus using CRISPR-Cas9 technology and inserted an EGFP-STOP cassette flanked by LoxP sites prior to the mutation site. The *Ifitm5^flox c.-14C>T^* mice develop normally. To induce the mutant MALEP-IFITM5 expression, we applied different Cre lines expressed in the skeleton at different developmental stages (osteochondral progenitors: *Prx1-Cre*; early osteogenic lineage: *Col1a1-Cre*; mature osteoblasts: *Ocn-Cre*). The *Prx1-Cre*; *Ifitm5^flox c.-14C>T^* compound mice presented severe skeletal deformities at embryonic and postnatal stages, while the *Col1a1-Cre* and *Ocn-Cre*; *Ifitm5^flox c.-14C>T^* compound mice showed milder phenotypes. Ectopic chondrogenesis was observed at the stage of primary spongiosa formation. Osteogenesis was significantly arrested, leading to long bone fractures at an early embryonic stage (E16.5). We isolated skeletal cells from the tibia of mutant mouse for single-cell RNA sequencing analyses at neonatal stage and identified a large population of “chondrocyte-like” osteoblasts expressing both osteogenic and chondrogenic markers. The mutant MALEP-IFITM5 may prime osteoblasts toward chondrocytes at early developmental stage and adipocytes in adult stage. This inducible *Ifitm5^flox c.-14C>T^* mouse model helps to gain deeper insight into the pathogenic mechanism of type V OI and provide new strategies for treating the disease.

## Materials and methods

### Ethical compliance

Animal care and experiments were conducted in accordance with the protocols approved by the Institutional Review Broad of the University of Hong Kong—Shenzhen Hospital ([2022]065). All the mouse lines used in this study were bred in Shenzhen Lingfu Tuopu Biotechnology Co., and maintained in equal split of day and night per day (Day: 7 am to 7 pm, Night: 7 pm to 7 am). The *Prx1-Cre* (JAX:005584) and *R26^tdTomato^* (Ai9, JAX:007909) mouse lines were obtained from Jackson laboratory. *Col1a1-Cre* (T004734) mouse line was purchased from Gempharmatech company. The *Ocn-Cre* (JAX:019509) mouse line was a gift from Dr. Chen Feng (The Third Hospital of Hebei Medical University, China). The *Ifitm5^flox-c.-14C>T^* knock-in mouse model was also generated by Gempharmatech company. The donor vector, guide RNA, insertion site, and primers used for genotyping were described in Table S1. All the mice were sacrificed by neck dislocation after anaesthetized. Phenotypes were compared between control and mutant groups consisting of both genders (biological replications ≥3 at different time-points). All the mutants analyzed in this study were compound heterozygous mice (*Cre*; *Ifitm5^flox-c.-14C>T^*), and control groups involved WT mice, mice with either *Cre* or *Ifitm5^flox-c.-14C>T^* allele.

### Skeletal preparation

The mice were eviscerated, fixed in 100% ethanol for 4 d, and transferred to acetone for 3 d. After rinsing with water, the skeletons were stained in Alcian blue/Alizarin red staining solution for 10 d (1 volume of 0.1% Alizarin red S in 95% ethanol, 1 volume of 0.3% Alcian blue 8GX in 70% ethanol, 1 volume of 100% acetic acid, and 17 volume of 100% ethanol). After being rinsed with water, the skeletons were kept in clearing solution (20% glycerol and 1% KOH) until clearly visible. The stained specimens were incubated in 50% and 80% (v/v) glycerol overnight for each solution and finally kept in 100% glycerol.

### Bone histology

The specimens were fixed in 4% paraformaldehyde and decalcified with 0.5 M EDTA before embedding in paraffin. The 6 μm sections were rehydrated and stained with Alcian blue and visualized with Leica DM3000 microscope.

### Immunostaining

The paraffin sections were dewaxed and rehydrated. Sections were blocked with blocking buffer (5% bovine serum albumin or donkey serum, 0.5% Tween20) for 1 h at room temperature. The primary antibodies of rabbit anti-SOX9 (Millipore, AB5535), rabbit anti-SP7 (Abcam, ab22552), goat anti-ALPL (R&D, AF2910), goat anti-SOST (R&D, AF1589), rabbit anti-PERILIPIN1 (Cell Signaling, 9349S), rabbit anti-RFP (Abcam, ab124754), and goat anti-RFP (Sicgen, AB8181) were diluted in blocking buffer and applied on the sections at 4 °C overnight. The signal was visualized by using donkey-anti-rabbit or donkey-anti-goat secondary antibodies and mounting with Vectashield mounting medium containing DAPI.

### Isolation of cells for single-cell transcriptomics

The same protocol was applied to isolate cells from OI_V mutant mouse as the control sample (GSE159544).^20^ Both tibiae were dissected from a *Prx1-Cre; Ifitm5^flox c.-14C>T^* mouse at P6 stage. Soft tissues were carefully removed, and bone tissues were cut into small pieces. Blood cells were digested away with TrypLE Express (Gibco) for 10 min. Then the bone was digested with type II collagenase (0.25%) and Dispase (0.25%) in HBSS at 37 °C for 1.5 h. Cells were collected every 30 min and filtered with a cell strainer (40 μm). Red blood cells were lysed using Red Blood Cell Lysis Solution (Miltenyi Biotec) and dead cells were removed with Dead Cell Removal Kit (Miltenyi Biotec). Viability of 82% was recorded. Total input of 10 000 cells was subjected for 10x sequencing. Single-cell encapsulation by Chromium single-cell platform (10X Inc.), library preparation using Single-Cell 3′ Reagent Kits v3, and paired-end sequencing (Illumina Novaseq 6000 platform) were performed in Berry Genomics Co.

### Single-cell RNA sequencing and bioinformatics analyses

The raw data of each sample were aligned to the mouse genome mm10. Then, data were further processed with the Seurat package (v5.0.3). For each dataset, cells with fewer than 1600 genes were filtered out. Doublets were predicted and removed using the DoubletFinder package (v2.0.4). Canonical correlation analysis was performed for dataset integration. Population signatures were identified by comparing each population against all other cells, using Seurat’s function “FindAllMarkers,” with an adjusted *p*-value <.05, log_2_FC > 1, and min.pct > 0.25. Chondrogenic (*Sox9*, *Acan*, *Col2a1*, *Col9a1*, and *Epyc*) and osteogenic (*Col1a1*, *Runx2*, *Bglap*, *Spp1*, and *Ifitm5*) populations were extracted for further analyses based on the signature genes, while the smooth muscle cells (*Tagln* and *Acta2*), endothelial cells (*Pecam1* and *Emcn*), neuronal cells (*Plp1* and *Sox10*), and hematopoietic lineage (*CD45*/*Ptprc*) were removed from the dataset after integrated analyses.

## Results

### Generation of an inducible mouse model (*Ifitm5*^*flox c.-14C>T*^) for type V OI

**Figure 1:**
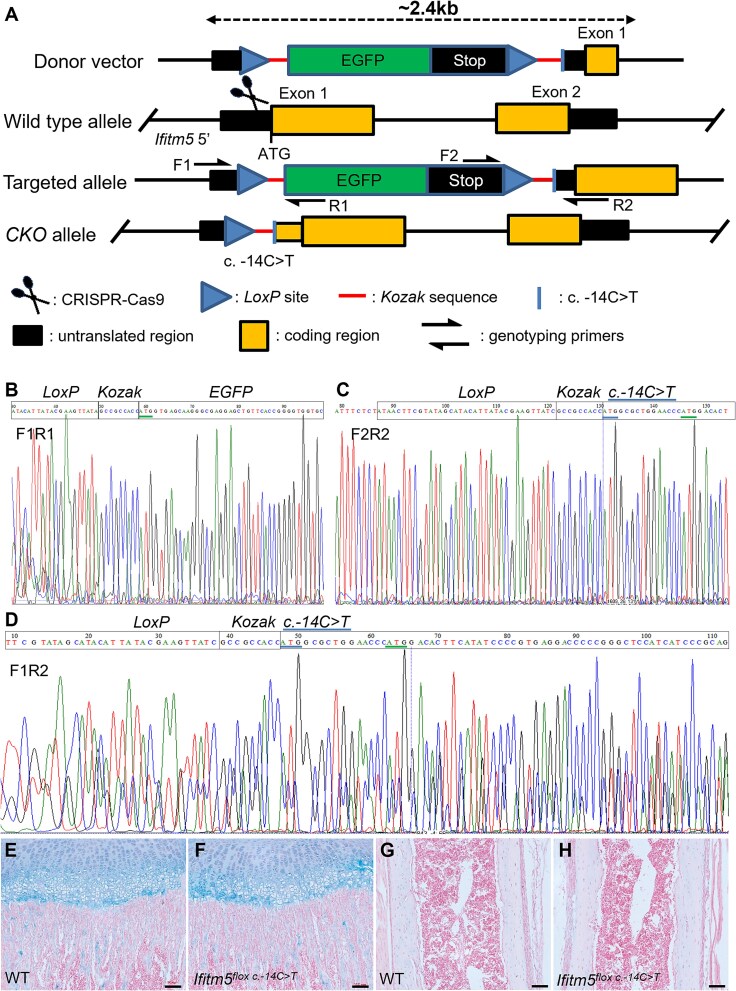
Generation of an inducible *Ifitm5* (c.-14C>T) mouse model. (A) Schematic diagram of targeting strategy. (B-C) Sanger sequencing of genotyping PCR products at 5′ (B, using F1R1 primers) and 3′ (C, using F2R2) ends of the inserted cassette. (D) Sanger sequencing of genotyping PCR product using F1R2 primers from *Prx1-Cre; Ifitm5^flox c.-14C>T^* mice. Both WT and mutant (with EGFP-Stop elements removed) allele could be detected. (E-H) Alcian blue staining of tibia growth plates (E-F) and cortical bone regions (G-H) from WT and *Ifitm5^flox c.-14C>T^* mice at P12 stage. Scale bar = 100 μm.

To avoid embryonic lethality of the *Ifitm5^c.-14C>T^* knock-in mice, we generated an inducible mouse model (*Ifitm5^flox c.-14C>T^*) using CRISPR-Cas9 technology in this study. The c.-14C>T mutation was induced in the *Ifitm5* locus and an *EGFP-STOP* cassette flanked by LoxP sites was inserted prior to the mutation site (Figure 1A, Table S1). Two independent founders were obtained. Genotyping using internal and external primers, and Sanger sequencing of the PCR products confirmed the insertion of *LoxP-EGFP-STOP-LoxP* cassette in the 5′ UTR before c.-14C>T mutation in the *Ifitm5* locus (Figure 1B and C). With Cre recombinase, the heterozygous *EGFP-STOP* cassette could be removed and mutant *Ifitm5^c.-14C>T^* could be transcribed (Figure 1D). The *Ifitm5^flox c.-14C>T^* mice and their offspring developed and grew normally. The histology of the growth plates (Figure 1E and F) and cortical bones (Figure 1G and H) of *Ifitm5^flox c.-14C>T^* mice was comparable with that of WT mice, consistent with previous study that *Ifitm5* loss-of-function had no significant effects on skeletal formation.^15^ We therefore used these mouse lines for skeletal characterization after mating with mice expressing Cre in the skeleton at different developmental stages.

### Skeletal phenotypes of the *Prx1-Cre; Ifitm5*^*flox c.-14C>T*^ compound heterozygous mice

In our previous study, we generated a single-cell transcriptome from mouse tibia at P6 stage (6 d old),^20^ showing the specific gene expression profiles in each population of chondrocytes and osteoblasts at different differentiation stages. Signature genes identified chondrogenic (*Acan*) and osteogenic (*Col1a1*) lineages (Figure S1A and B). *Lepr* and *Bglap* (*Osteocalcin*) mark the osteogenic progenitors and mature osteoblasts, respectively (Figure S1C and D). The expression pattern of *Ifitm5* suggests its expression in the mature osteoblasts (Figure S1E). As *Prrx1* starts to express during the limb mesenchymal condensation,^21^ to remove *EGFP-STOP* cassette and induce mutant *Ifitm5* (*Ifitm5^c.-14C>T^*) expression, we utilized various mouse lines expressing Cre at different stages during skeletal development, including *Prx1-Cre* (skeletal progenitors), *Col1a1-Cre* (osteogenic lineage), and *Ocn-Cre* (mature osteoblasts).

**Figure 2:**
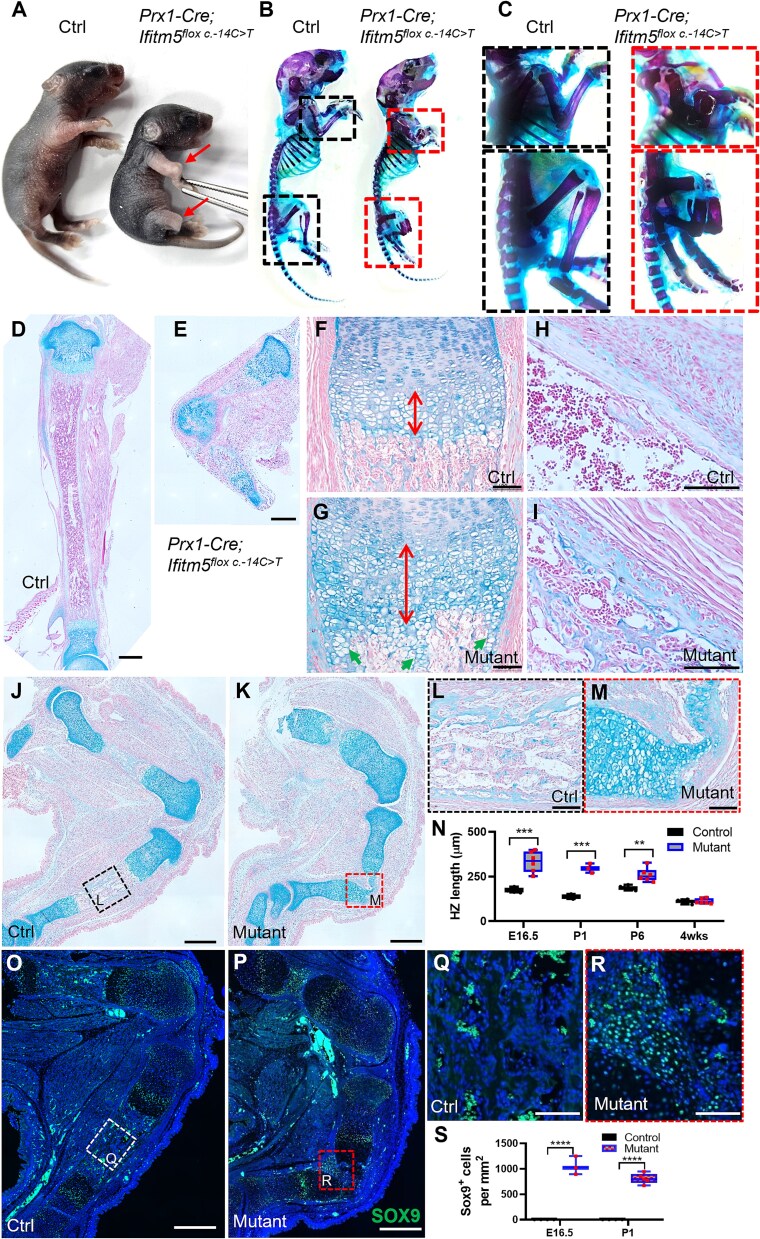
Abnormal bone development in *Prx1-Cre; Ifitm5^flox c.-14C>T^* mice. (A) Gross appearance of control (*Ifitm5^flox c.-14C>T^*) and mutant (*Prx1-Cre; Ifitm5^flox c.-14C>T^*) mice at P6 (6-d-old) stage. Fractures in the forelimb and hindlimb of mutant mouse were arrowed. (B-C) Skeletons of control and mutant mice stained with Alcian blue and alizarin red at P6 stage. Boxed regions were shown at higher magnification. (D-E) Alcian blue staining of the tibia from control and mutant mice at P6 stage. (F-I) The distal growth plates and cortical bone regions from control and mutant mice at P6 stage were shown at higher magnification. Cartilaginous remnants were indicated by arrow heads (G). (J-M) Alcian blue staining of femur and tibia sections from control and mutant mice at E16.5 stage. The diaphysis regions of tibia were shown at higher magnification. (N) Quantification of the length of hypertrophic zone (HZ) from control and mutant groups at different stages. The length at the middle line of the HZ was indicated by the arrows. The averaged length with standard deviation (*n* ≥ 3) was presented in bar charts. (O-R) Immunostaining of SOX9 on the tibia sections from WT and mutant mice at E16.5 stage. The boxed cortical bone regions were shown at higher magnification. (S) Quantification of the SOX9^+^ cells in the diaphysis regions from control and mutant groups at different stages. The averaged cell density with standard deviation (*n* ≥ 3) was presented in bar charts. *p*-values were calculated by Student *t*-test, 2-tailed, unpaired. **: *p*-value <.01; ***: *p*-value <.001; ****: *p*-value <.0001. Scale bar in D-E, J-K, and O-*P*=500 μm; scale bar in F-I, L-M, and Q-R = 100 μm.

The *Prx1-Cre; Ifitm5^flox c.-14C>T^* compound mice can survive to adulthood. We observed normal Mendelian ratio of the offsprings (26.9 ± 8.29%, No. of total mutants ≥22, including 3-mo-old mutants). However, the mutant mice were born with fractures in both forelimbs and hindlimbs (Figure 2A). Skeletal preparation showed normal craniofacial structure, ribcage and axial skeleton, but fractures in the tibia-fibula and radial-ulna bones (Figure 2B and C). To explore the molecular impact of mutant *Ifitm5^c.-14C>T^* (MALEP-IFITM5) on bone development, we compared the bone histology between *Prx1-Cre; Ifitm5^flox c.-14C>T^* compound mutants and control littermates at P6 stage. Surprisingly, a cartilaginous callus was observed in the diaphysis bone region of the mutant tibia (Figure 2D and E). Similar deformity was also found in the femur of the mutant mice (Figure S2A and B). Although Ifitm5 was not expressed in chondrocytes, the hypertrophic zone in the growth plate was significantly expanded, and cartilaginous remnants were present in the trabecular region (Figure 2F and G). The cortical bone was disorganized with increased cell density embedded in the bone matrix (Figure 2H and I).

To examine bone phenotypes at the embryonic stage, we analyzed the bone histology of littermates at E18.5. In addition to the tibia, we also found cartilaginous callus in the femur (Figure S2C-H). During endochondral formation, primary spongiosa forms at around E15.5 in the tibia, coupled with vascular invasion and osteoblast differentiation.^22^ We further analyzed whether the skeletal defects occurred at the beginning of the primary ossification center. In the control mice, the proximal and distal tibial growth plates were separated with primary spongiosa forming at E16.5 (Figure 2J and L). However, continuous growth plate cartilage was observed in mutant mice (Figure 2K and M). The expansion of hypertrophic zone was also observed in the embryonic stages (Figure 2N). Before the primary spongiosa formed (E14.5), the morphologies of control and mutant mice were comparable (Figure S3A). No obvious difference was found in the histology of tibia and femur at this stage (Figure S3B and C). These data suggest that the skeletal defects in the *Prx1-Cre; Ifitm5^flox c.-14C>T^* mutants may occur when the primary spongiosa starts to form.

### Ectopic chondrogenesis in the diaphysis region of *Ifitm5* mutant mice

To explore the molecular signature of the cartilaginous callus, we examined the expression patterns of the key regulators during endochondral bone formation. SOX9 is the master regulator that converts progenitor cells into chondrocytes and regulates the sequential stages of chondrocyte differentiation.^23^ In control mice at newborn stage, SOX9 is mainly expressed in proliferating chondrocytes, but not in hypertrophic chondrocytes and osteoblasts in the cortical bone region (Figure S2I, K, and M). In mutant mice, we observed a similar expression pattern of SOX9 expression in the growth plate region (Figure S2J and L). However, a large number of SOX9^+^ chondrocytes were found in the diaphyseal region, confirming the chondrogenic fate of these abnormal cells (Figure S2N). We then investigated whether abnormal chondrogenesis occur in embryonic stage. Before the primary spongiosa formed, the control and mutant mice showed similar expression patterns of SOX9 in the limbs at E14.5 stage (Figure S3D-G). At E16.5, SOX9^+^ cells were accumulated in the diaphysis region of both femur and tibia of mutant mice (Figure 2O-R). The numbers of SOX9^+^ cells were significantly increased in the cortical bone regions of the mutant mice at embryonic (E16.5) and postnatal (P1) stages (Figure 2S), which demonstrated that the mutant IFITM5 induced ectopic chondrogenesis in the diaphysis during the early development of primary ossification center.

### Analyses of single-cell transcriptomics revealed chondrocyte-like osteoblasts in *Prx1-Cre; Ifitm5*^*flox c.-14C>T*^ mice

**Figure 3:**
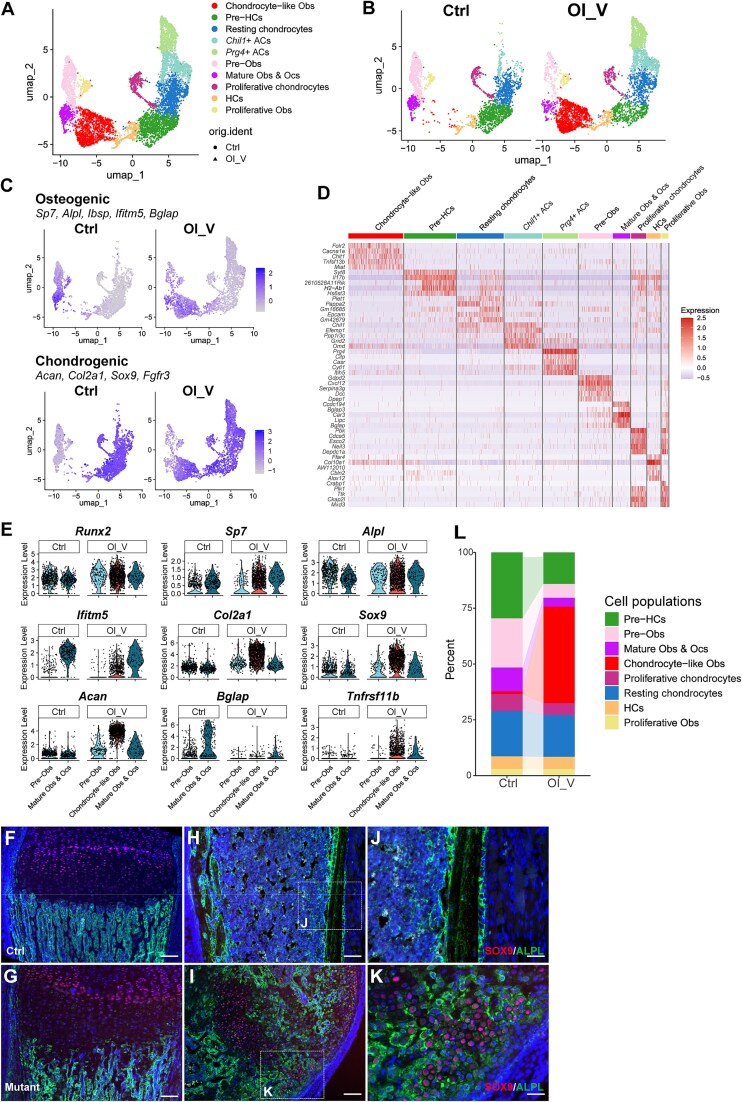
Single-cell transcriptomic analyses of the tibiae from control (ctrl) and *Prx1-Cre; Ifitm5^flox c.-14C>T^* (OI_V) mice at P6 stage. (A-B) UMAP plot showing annotated clusters of the skeletal cells from integrated (A) and separated samples (B). Obs: osteoblasts; Ocs: osteocytes; ACs: articular cartilage cells; HCs: hypertrophic chondrocytes. (C) Feature plots showing the lineage scores of the osteogenic and chondrogenic cell populations in each sample. (D) Heatmap showing the normalized expression levels of the top 5 differentially expressed genes in each cluster. (E) Violin plot showing the expression patterns of the selected feature genes in different osteogenic populations in each sample. Cells marked as “chondrocyte-like Obs” in the control sample was not included due to the limited cell number. (F-K) Co-immunostaining of SOX9 (red) and ALPL (green) on the tibia sections from WT and mutant mice at P6 stage. The boxed cortical bone regions were shown at higher magnification. Scale bar = 100 μm. (L) Relative proportion of cell populations in the skeletal lineage. Articular chondrocytes were excluded as the articular cartilages were removed before the single cell isolation in the control sample.

To investigate the underlying mechanisms of cell-fate change in the *Ifitm5* c.-14C>T mutant mice, we performed single-cell transcriptomics (scRNA-seq) on the tibiae of *Prx1-Cre; Ifitm5^flox c.-14C>T^* (OI-V) and the control mice^20^ at P6 stage. Following the standard protocol of quality assessment, 8226 and 8867 cells from control and OI-V samples were collected, respectively. Integrated clustering analyses of both samples identified 12 clusters (Figure S4A and B). From their molecular signatures, osteogenic cells (*Col1a1*, *Runx2*, *Bglap*, *Spp1*, and *Ifitm5*), chondrocytes (*Sox9*, *Acan*, *Col2a1*, *Col9a1*, and *Epyc*), ligament and tendon cells (*Tnmd* and *Aspn*), smooth muscle cells (*Tagln* and *Acta2*), endothelial cells (*Pecam1* and *Emcn*), neuronal cells (*Plp1* and *Sox10*), and hematopoietic lineage (*CD45*/*Ptprc*) were identified (Figure S4C and D, Table S2). Chondrogenic and osteogenic populations were extracted for further analyses based on a combination of signature markers (Figure S4D). A total of 2933 and 4497 cells were isolated from the control and *Prx1-Cre; Ifitm5^flox c.-14C>T^* samples, respectively. Integrated clustering analysis identified 10 populations in the mixed samples (Figure 3A and B). Expression patterns of specific markers helped to annotate each population (Figure 3C, Table S2). We compared the chondrogenic populations and signature genes between the control and mutant mice, and identified similar patterns and profiles of resting chondrocytes, proliferating chondrocytes, pre-hypertrophic chondrocytes, and hypertrophic chondrocytes (Figure 3B and D). In the control sample, the epiphyseal articular cartilage was removed before single-cell isolation.^20^ As a previous study indicated mutant IFITM5 caused ossification in the knee and ankle, the whole tibia including the epiphysis from the mutant mouse was subjected for digestion at P6 stage, explaining why a large population of articular cartilage cells was observed in the mutant sample (Figure 3B and C). However, no *Ifitm5* expression was detected in the articular chondrocytes and these cells were clustered closed to the growth plate chondrocytes (Figure 3A-C, Table S2). Focusing on the osteogenic lineage, 3 clusters were identified at different differentiation stages, including proliferative osteoblasts (*Top2a* and *Mki67*), pre-osteoblasts (*Cxcl12* and *Lepr*), mature osteoblasts, and osteocytes (*Ifitm5, Dmp1*, and *Phex*). Interestingly, in the *Prx1-Cre; Ifitm5^flox c.-14C>T^* sample, an aberrant cell group was identified in addition to the 3 osteogenic clusters mentioned previously (Figure 3D). In the control sample, the skeletal cells express either osteogenic gene (*Alpl*, *Ibsp*, *Ifitm5*, and *Bglap*) or chondrogenic gene (*Acan*, *Col2a1*, *Sox9*, and *Fgfr3*). However, the aberrant cell cluster expressed both osteogenic and chondrogenic markers (Figure 3D and E, Figure S5), leading to the term “chondrocyte-like osteoblasts.” To validate these special cell populations, we performed co-immunostaining using SOX9 and ALPL antibodies on the tibia sections. In the growth plates of control and mutant mice, we found SOX9 was expressed in the resting and proliferating chondrocytes, and ALPL was expressed in the trabecular regions (Figure 3F and G). In the cortical bone of control mice, ALPL was mainly expressed in the mature osteoblasts, and no SOX9^+^ cells were detected (Figure 3H and J). Interestingly, in the diaphysis region of the mutant tibia, SOX9^+^/ALPL^+^ chondrocyte-like osteoblasts were found in addition to the SOX9^+^ chondrocytes and ALPL^+^ osteoblasts (Figure 3I and K). Based on the quantification of scRNA-seq data, these chondrocyte-like osteoblasts accounted for a large proportion of cells in the mutant sample. As a result, the percentage of mature osteoblasts was significantly lower in the mutant sample (Figure 3L).

### Compromised osteoblast differentiation and maturation in *Prx1-Cre; Ifitm5*^*flox c.-14C>T*^ mice

**Figure 4:**
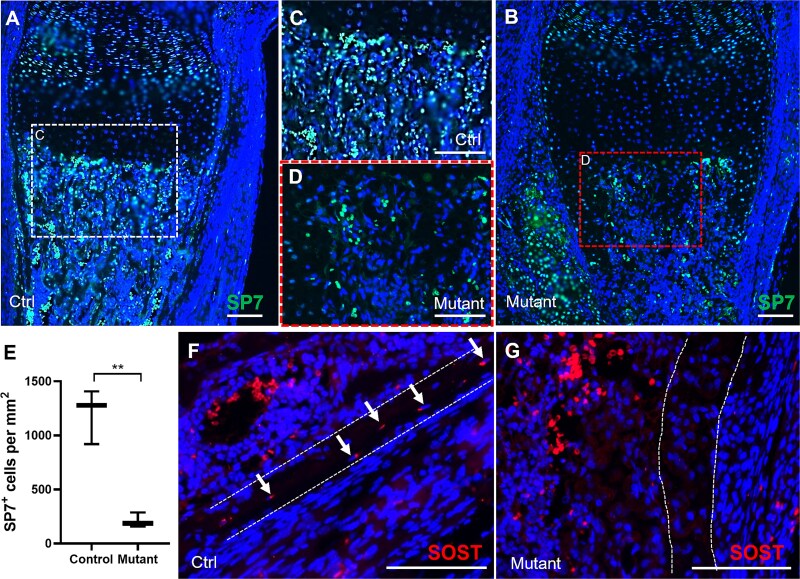
Aberrant osteogenesis in *Prx1-Cre; Ifitm5^flox c.-14C>T^* mice. (A-D) Immunostaining of SP7 on proximal tibia regions of tibia sections from control and mutant mice at P1 stage. The boxed trabecular bone regions were shown at higher magnification (C-D). (E) Quantification of the SP7^+^ cells in the trabecular regions (0.2 mm underneath the chondro-osseous junction) from the proximal tibia of control and mutant mice at P1 stage. The averaged cell density with standard deviation (*n* ≥ 3) was presented in bar charts. *p*-values were calculated by Student *t*-test, 2-tailed, unpaired. **: *p*-value <.01. (F-G) Immunostaining of SOST on cortical bone regions of tibia sections from control and mutant mice at P1 stage. SOST^+^ osteocytes were arrowed. Scale bar = 100 μm.

Single-cell transcriptomic analyses have revealed abnormal osteoblast differentiation with mutant *Ifitm5* expression. The presence of chondrocyte-like osteoblasts may compromise the normal bone formation with the expense of osteocyte maturation. The expression level of mature osteoblast marker *Bglap* (Osteocalcin) was significantly down-regulated in the osteogenic populations of mutant mice (Figure 3E). To further validate, we examined the expression of SP7, an osteoblast-specific transcription factor on the tibia sections. In the control mice, SP7 was expressed in prehypertrophic chondrocytes and osteoblasts in the trabecular region (Figure 4A). Consistently, SP7 was detected in the prehypertrophic region of mutant mice, though the hypertrophic zone was expanded (Figure 4B). However, in the trabecular bone, the SP7^+^ osteoblasts were dramatically reduced, as compared with the control group (Figure 4C-E).

During bone growth, osteoblasts differentiate into osteocytes that are embedded in the calcified bone matrix. Osteocytes secret Sclerostin (SOST) to inhibit WNT signaling and maintain bone homeostasis.^24^ To test whether osteocyte maturation was affected in mutant mice, we compared the expression levels of SOST. In the cortical bone of control mice, SOST was expressed in the spindle-shaped osteocytes embedded in well-organized bone matrix (Figure 4F). In mutant mice, the cells in the cortical bone were more rounded and in higher density, and no SOST was detected in these cells (Figure 4G), suggesting that osteocyte maturation was arrested during the skeletal development of *Prx1-Cre; Ifitm5^flox c.-14C>T^* mutant mice.

### Enhanced marrow adipogenesis in the adult *Prx1-Cre; Ifitm5*^*flox c.-14C>T*^ mutant mice

**Figure 5:**
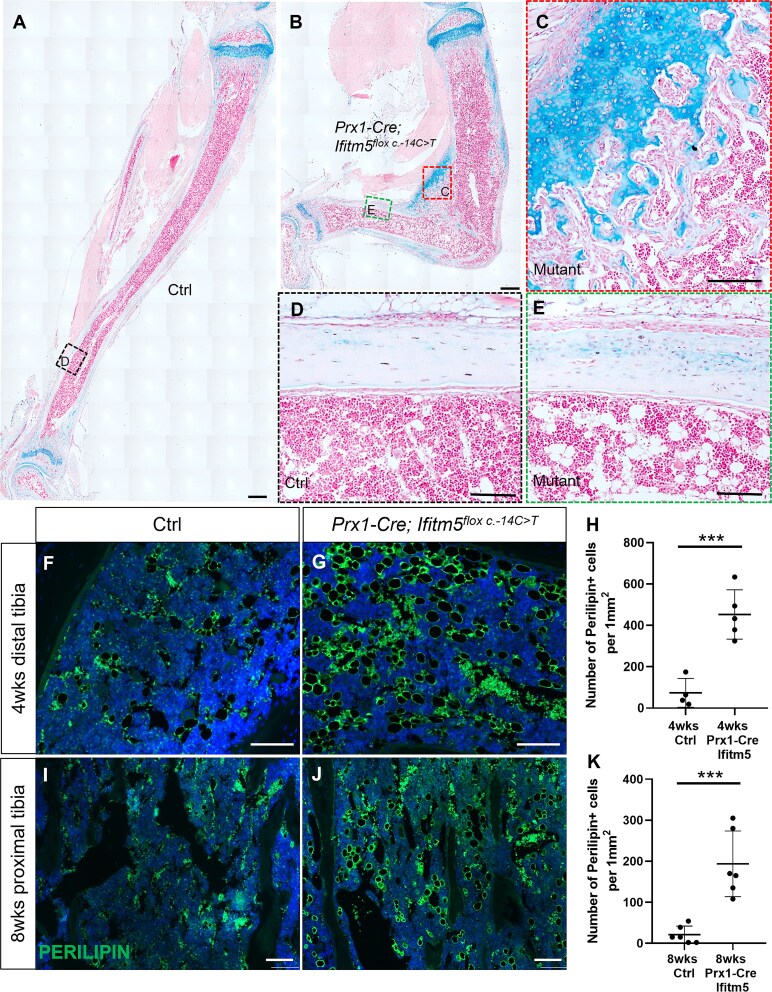
Enhanced marrow adipogenesis in adult *Prx1-Cre; Ifitm5^flox c.-14C>T^* mice. (A-E) Alcian blue staining of the tibia from control and mutant mice at 4-wk stage. The boxed cortical bone regions were shown at higher magnification. Scale bar in A-B = 500 μm; scale bar in C-E = 100 μm. (F-G) Immunostaining of PERILIPIN on the distal tibia sections from control and mutant mice at 4-wk stage. Scale bar = 100 μm. (H) Quantification of PERILIPIN^+^ adipocytes in marrow space of WT and mutant mice at 4-wk stage. (I-J) Immunostaining of PERILIPIN on proximal tibia sections from control and mutant mice at 8-wk stage. Scale bar = 100 μm. (H) Quantification of PERILIPIN^+^ adipocytes in marrow space of the control and mutant mice at 8-wk stage.

To follow up the remodeling process of the cartilaginous callus, we analyzed the bone phenotypes of the littermates at adult stages (Figure S6A-E). The tibia of *Prx1-Cre; Ifitm5^flox c.-14C>T^* mutant mice at 4 wk old of age remained deformed, while the length of hypertrophic zones became comparable (Figures 5A, B and 2N). A large number of chondrocytes were present in the diaphyseal region (Figure 5C). Consistently, cell density was significantly higher in mutant cortical bone as compared with control mice, and cell orientation was disorganized (Figure 5D and E), which was similar with the bone phenotype found in type V OI patients.^25^ Interestingly, we noticed there were more adipocytes in the distal tibial marrow cavity of mutant mice (Figure 5E). Staining with the adipocyte marker PERILIPIN showed that more adipocytes were present in the distal tibia of *Ifitm5* mutants (Figure 5F-H). Regarding the difference of adipocyte development between the proximal and distal ends of tibia, more adipocytes were observed in the proximal tibia of *Ifitm5* mutants at 8-wk stage, but hardly detected in the control sample (Figure 5I-K). Milder enhanced adipogenesis was also observed in the femur at this stage (Figure S6F and G). Since osteoblasts and adipocytes could be derived from the same mesenchymal progenitors, we asked whether the increased adipocytes were the descendants of *Prrx1* expressing progenitors. Co-staining of PERILIPIN and tdTomato on the tibia section showed that the majority of PERILIPIN^+^ adipocytes in the mutant mice were tdTomato positive (Figure S6H and I). However, whether these adipocytes are transdifferentiated from osteoblasts that ever express mutant *Ifitm5* requires further investigation. To further characterize the impact of mutant IFITM5 on the differentiation potential of skeletal progenitors, we isolated the marrow mesenchymal stem cells (MSCs) from the control and mutant tibia at P6 stage for *in vitro* differentiation. Although we did not observe obvious difference of the osteogenic potential between the control and mutant MSCs (Figure S7A-C), the chondrogenesis (Figure S7D and E) and adipogenesis (Figure S7F-J) were significantly enhanced in the mutant MSCs when compared with the control cells, consistent with the *in vivo* phenotypes observed.

Bone formation is tightly coupled with bone resorption during early skeletal development. Thus, we examined the difference of osteoclasts between the control and mutant mice by tartrate-resistant acid phosphatase (TRAP) staining. In the trabecular regions, the TRAP signals were comparable between the control and mutant mice (Figure S7K, L, and R). Few osteoclasts were detected in the well-organized cortical bones (Figure S7M and N). Interestingly, significantly more TRAP^+^ osteoclasts were found in the malformed diaphysis region of the mutant tibia, when comparing with the control mice (Figure S7O-R), suggesting active bone remodeling in this region.

### Skeletal phenotypes of *Col1a1-Cre*/*Ocn-Cre; Ifitm5*^*flox c.-14C>T*^ compound heterozygous mice

**Figure 6:**
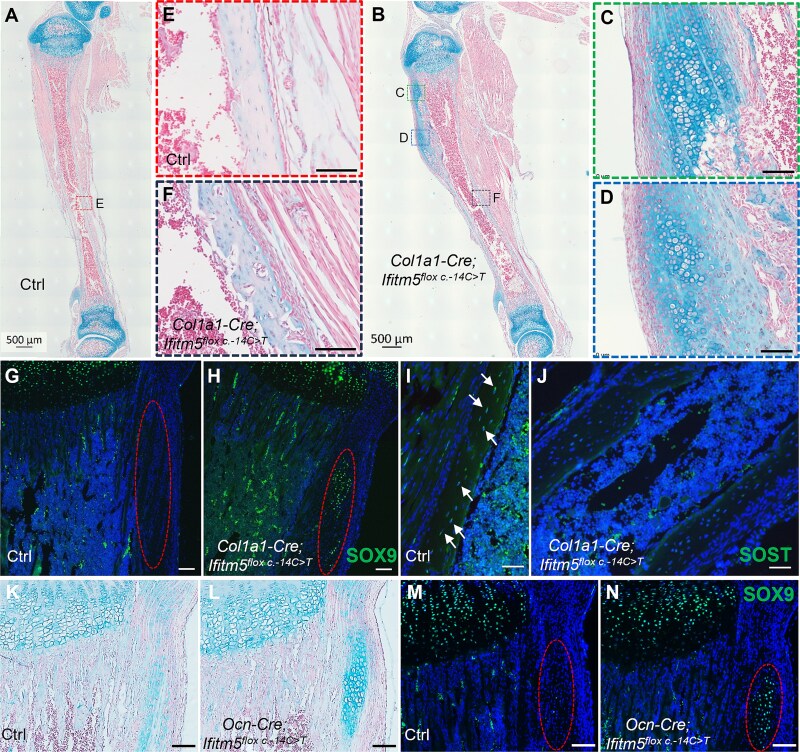
Skeletal phenotypes in *Col1a1-Cre; Ifitm5^flox c.-14C>T^* and *Ocn-Cre; Ifitm5^flox c.-14C>T^* mice. (A-F) Alcian blue staining of the tibia from control and *Col1a1-Cre; Ifitm5^flox c.-14C>T^* mice at P10 stage. The boxed cortical bone regions were shown at higher magnification. Scale bar in A-B = 500 μm; scale bar in C-F = 100 μm. (G-H) Immunostaining of SOX9 on the tibia sections from control and *Col1a1-Cre; Ifitm5^flox c.-14C>T^* mice at P10 stage. (I-J) Immunostaining of SOST on the tibia sections from control and *Col1a1-Cre; Ifitm5^flox c.-14C>T^* mice at P10 stage. SOST^+^ osteocytes were arrowed. (K-L) Alcian blue staining of the tibia from control and *Ocn-Cre; Ifitm5^flox c.-14C>T^* mice at P10 stage. (M-N) Immunostaining of SOX9 on the tibia sections from control and *Ocn-Cre; Ifitm5^flox c.-14C>T^* mice at P10 stage. Scale bar in G-N = 100 μm.

*Prrx1* marks all the mesenchymal progenitors that contribute to chondrocytes, osteoblasts, and adipocytes, whereas *Col1a1-Cre* marks mainly osteoblasts in bone. To explore the impact of mutant IFITM5 on the osteogenic lineage, we analyzed the skeletal phenotypes in *Col1a1-Cre; Ifitm5^flox c.-14C>T^* compound mice. The skeletal morphologies of skull, rib cage, spine, and long bone were comparable between the control and mutant littermates (Figure S8A-I). We did not observe long bone fractures in these mutants (Figure 6A and B). However, ectopic chondrogenesis was observed in the bone collar regions of the mutant mice (Figure 6C and D). Osteogenesis was also attenuated, with higher porosity and cell density in the cortical bone of mutant mice compared with control mice (Figure 6E and F). SOX9 was ectopically expressed in the cartilaginous callus in the bone collar region of the mutant mice (Figure 6G and H). The expression of SOST was relatively low in the mutant cortical bone (Figure 6I and J), suggesting that osteocyte maturation was arrested in the *Col1a1-Cre; Ifitm5^flox c.-14C>T^* compound mice.

*Osteocalcin* (*Bglap*) showed similar expression pattern to *Ifitm5* in normal mice (Figure S1). Thus, removal of the EGFP-STOP cassette by *Ocn-Cre* may have delayed effects. The *Ocn-Cre; Ifitm5^flox c.-14C>T^* compound mice were grossly normal with respect to body size and skeletal length. Close examination of the skeleton revealed only mild ectopic chondrogenesis in the bone collar of the proximal tibia of *Ocn-Cre; Ifitm5^flox c.-14C>T^* mice (Figure 6K and L). Immunostaining confirmed the SOX9 expression in this area (Figure 6M and N).

## Discussion

The perinatal lethality of the knock-in (*Ifitm5^c.-14C>T^*)^17^ and transgenic (*Col2.3*-Mut *Ifitm5*)^16^ mice hampered the evaluation of the bone parameters at postnatal stages and identification of therapeutic targets. This study presented a novel inducible *Ifitm5^flox c.-14C>T^* mouse model that could be modulated by Cre expressing at different developmental stages under the regulation of its endogenous regulatory elements. Similar to the previous finding using the conditional *Rosa26^mIfitm5^* mouse model,^19^ induction of the mutant IFITM5 in the skeletal progenitors by *Prx1-Cre* caused severe bone deformities, impaired endochondral ossification, and cartilage overgrowth at early developmental stage. The *Rosa26^mIfitm5^*Prx1-Cre mice showed normal cortical thickness and morphology,^19^ while in our study ectopic growth of cartilage in the diaphysis regions and bending of long bones were observed in the *Prx1-Cre; Ifitm5^flox c.-14C>T^* mice (Figure 2D and E). Overexpression of mutant IFITM5 in the chondrocytes mainly resulted in abnormal growth plate architecture, reduced hypertrophic zone, and progressive joint deformity.^19^ However, in the *Prx1-Cre; Ifitm5^flox c.-14C>T^* mice, the hypertrophic zone was expanded with cartilaginous remnants in the trabecular region (Figure 2F and G). These dichotomous results may be resulted from distinct spatiotemporal expression pattern of the mutant IFITM5. Expression of the mutant IFITM5 in the committed (*Rosa26^mIfitm5^*Col1a1-Cre) or mature (*Rosa26^mIfitm5^*Ocn-Cre) osteoblasts did not show obvious effect on the skeletal development,^19^ while in the *Col1a1-Cre* and *Ocn-Cre; Ifitm5^flox c.-14C>T^* mice ectopic cartilage formation was still observed in the periosteum region (Figure 6), though the phenotypes were much milder. The different severity of the compound mice (*Prx1-Cre*, *Col1a1-Cre*, and *Ocn-Cre; Ifitm5^flox c.-14C>T^*) also confirmed the feasibility of this mouse model to examine the different response of skeletal progenitors, immature and mature osteoblasts to the mutant IFITM5 protein. These compound mice could survive until adulthood, making it possible to explore the impact of mutant IFITM5 on bone homeostasis at adult stage and screen potential therapeutic targets for type V OI.

The presence of cartilaginous remnants in the trabecular region, ectopic chondrogenesis in the diaphysis, and enhanced marrow adiposity of the *Prx1-Cre; Ifitm5^flox c.-14C>T^* mice suggest cell fate changes of the progenitors toward osteogenesis, chondrogenesis, and adipogenesis. The homeostasis coordinating the cell fate determination of skeletal progenitors in these mutant mice should become imbalanced, yet the underlying regulatory network still requires further investigation. Lineage tracing studies have demonstrated that hypertrophic chondrocytes can become osteoblasts and contribute to bone formation and fracture healing.^26^^,^^27^ Previous analyses of single-cell RNA-seq data identified a population of chondrocyte-like osteoprogenitors (COPs) expressing both chondrogenic (*Sox9* and *Acan*) and osteogenic (*Runx2* and *Sp7*) markers.^28^ These COPs could arise from hypertrophic chondrocytes and perichondrium, supplying trabecular osteoblasts and marrow stromal cells during bone development. It is still uncertain about the difference and similarity of the COPs and chondrocyte-like osteoblasts identified in our study. It would be worth for further exploration that whether the mutant IFITM5 changes the fate of COPs in the mutant mice, and whether the expansion of hypertrophic zone, remnants of chondrocytes in the diaphysis, and existence of chondrocyte-like osteoblasts may reflect the defect of chondrocyte-osteoblast transformation.

According to the single-cell RNA-seq data from mouse tibia (P6)^20^ and spine (P10 and 8-wk),^29^  *Ifitm5* was mainly expressed in the mature osteoblast but not chondrogenic lineage. However, we observed exuberant cartilage formation in the compound mutant mice. Induction of mutant IFITM5 in the growth plate by Acan-CreERt2 also led to enhanced chondrogenesis.^19^ During endochondral ossification, hypertrophic chondrocytes start to express matrix metalloproteinases (MMP9, MMP13, mainly regulated by Osterix and Runx2) to cleavage and degrade cartilage matrix, coupling with vascular invasion and osteogenesis to form bone cortex.^30–34^ The absence of metalloproteinases causes delayed endochondral ossification and expanded hypertrophic zone in both *Osx^-/-^* and *Mmp13^-/-^* mice.^35^^,^^36^ In *Mmp13* null mice, the enlarged hypertrophic zone persisted until maturity and gradually returned to normal size, probably due to compensation by other metalloproteinases.^36^ The *Prx1-Cre; Ifitm5^flox c.-14C>T^* mice showed expanded hypertrophic zones and delayed matrix resorption at early stage, and displayed normal growth plate phenotypes at 4-wk-old stage, resembling the phenotypes of *Mmp9* and *Mmp13* null mice.^34^^,^^36^ There may be a link between the mutant protein and reduced activities of matrix collagenases, although this is not yet confirmed. Moreover, we observed more TRAP^+^ osteoclasts in the cartilaginous callus regions of the mutant mice. However, we found the expression of OPG/Osteoprotegerin (*Tnfrsf11b*, Figure 3E) was up-regulated in the mutant osteoblasts. OPG is a decoy receptor for RANKL and inhibits the differentiation of osteoclasts.^37^ Whether the osteoclasts are involved in the bone phenotypes of *Ifitm5* mutant mice and type V OI patients deserves further investigation.

**Figure 7:**
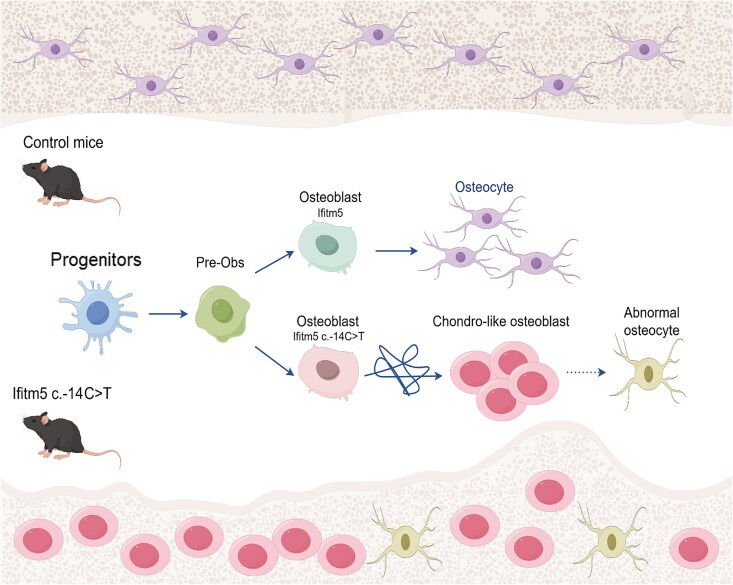
Schematic representation of aberrant osteogenic differentiation in *Ifitm5 c.-14C>T* mice. In normal mice, progenitors differentiate to osteogenic lineage and mature into osteocytes embedded in the bone matrix. In *Ifitm5 c.-14C>T* mice, osteoblasts expressing MALEP-IFITM5 differentiate into chondrocyte-like osteoblasts, and the maturation of osteocytes becomes compromised. Figure was drawn using Figdraw. Pre-Obs: Pre osteoblasts.

Postnatal and adult skeletal examination provided more clues to the pathogenesis of the mutant IFITM5 protein. The presence of cartilaginous callus in the diaphysis of adult *Prx1-Cre; Ifitm5^flox c.-14C>T^* mice, and ectopic chondrogenesis in the perichondrium and periosteum regions of *Col1a1-Cre and Ocn-Cre; Ifitm5^flox c.-14C>T^* mice suggest altered differentiation trajectory of osteo-chondroprogenitors by mutant IFITM5. Findings in the *Rosa26-Ifitm5* (c.-14C>T) mouse model also pointed to elevated SOX9 level and activation of ERK/MAPK signaling.^19^ MSCs are known to have the potential to differentiate into osteogenic, adipogenic, and chondrogenic lineages. Each of these events is highly regulated and exclusive, meaning that commitment to 1 lineage would have an inhibitory effect on the others. The commitment of mesenchymal cells into chondrocytes, osteoblasts, and adipocytes is coordinated by multiple transcription factors and signaling pathways, including SOX9, RUNX2, β-CATENIN, and PPARγ.^38–40^ High level of WNT/β-CATENIN signaling is required for induction of osteogenesis likely through increasing *Runx2* expression, but inhibition of chondrogenesis and adipogenesis via repression of SOX9 and PPARγ, respectively.^41–44^  *In vitro* studies demonstrated increased expression of adipocytic markers *AdipoQ* and *Fabp4* in primary osteoblasts isolated from *Ifitm5^c.-14C>T^* knock-in mice.^17^ In this study, we also observed compromised osteoblast maturation (reduced expression of SP7, target of RUNX2^45^) and elevated adipocytes in the marrow cavity of adult *Prx1-Cre; Ifitm5^flox c.-14C>T^* mice, indicating mutant MALEP-IFITM5 disrupted the homeostasis of chondrogenic, osteogenic, and adipogenic fates of mesenchymal progenitors. Interestingly, our scRNA-seq data revealed the chondrocyte-like osteoblast population from the mutant mice displayed bipotent characteristics, expressing both osteogenic and chondrogenic markers. The homeostatic regulation of cell fate determination in skeletal progenitors in these mutant mice might be imbalanced (Figure 7), yet the underlying regulatory network still requires further investigation. Future *in vivo* study evaluating the possible transdifferentiation of osteoblasts into chondrocytes will be of interest.

The compound mice exhibited significant skeletal phenotypes, indicating a pathogenic role of *Ifitm5^c.-14C>T^* in skeletal development. The skeletal features observed in the mice, such as high bone cell density, porous bone, and intrauterine long bone fracture, partially replicate the clinical features in human. However, the mice did not exhibit the characteristic manifestations of hyperplastic callus formation, interosseous membrane calcification, or radial head dislocation observed in type V OI patients.^10^^,^^11^^,^^25^^,^^46^ Although the mechanism behind the development of these distinctive phenotypes remains unclear, the regulation of fate commitment of the skeletal progenitors in type V OI patients during development and fracture conditions may be impaired.

In summary, we presented an inducible mouse model (*Ifitm5^flox c.-14C>T^*) that could be induced by specific Cre with endogenous regulatory elements maintained. *Prx1-Cre; Ifitm5^flox c.-14C>T^* mutant mice showed severe bone deformity, compromised osteogenesis, and enhanced chondrogenesis associated with ectopic SOX9 expression. Analyses of single-cell RNA sequencing data identified a chondrocyte-like osteoblast population expressing both osteogenic and chondrogenic signature genes in *Prx1-Cre; Ifitm5^flox c.-14C>T^* mouse. The severity of skeletal abnormality was reduced in *Col1a1-Cre/Ocn-Cre; Ifitm5^flox c.-14C>T^* mutant mice, while ectopic chondrogenesis was still observed in the perichondrium regions. Therefore, this inducible *Ifitm5^flox c.-14C>T^* mouse line could be a useful model to gain better insights into the pathogenic mechanisms of OI type V, as well as aiding the development of new therapeutic strategies.

## Supplementary Material

Ifitm5_mouse_paper_JBMR_SupFigures_20250114_zjaf022

Table_S1_Inducible_Ifitm5_Knock-in_design_zjaf022

Table_S2_Top_20_signature_genes_in_each_population_zjaf022

## Data Availability

The single-cell RNA sequencing data for the control sample were downloaded from the GEO database (GSE159544). The single-cell transcriptomic data from the *Prx1-Cre; Ifitm5^flox c.-14C>T^* mouse at P6 stage were deposited on the NCBI GEO website (GSE264540). Scripts for analyzing the data were deposited at https://github.com/LIZELUAN/P6_Mouse_tibia_IFITM5_vs_Ctrl.
