# Supplementary material for: An inducible mouse model of osteogenesis imperfecta type V reveals aberrant osteogenesis caused by Ifitm5 c.-14C>T mutation
Source: J Bone Miner Res. 2025 Feb 5;40(5):577–90. doi: 10.1093/jbmr/zjaf022 (PMC12103725; doi:10.1093/jbmr/zjaf022)
Supplement: Ifitm5_mouse_paper_JBMR_SupFigures_20250114_zjaf022 [file ifitm5_mouse_paper_jbmr_supfigures_20250114_zjaf022.pdf]

Fig. S1

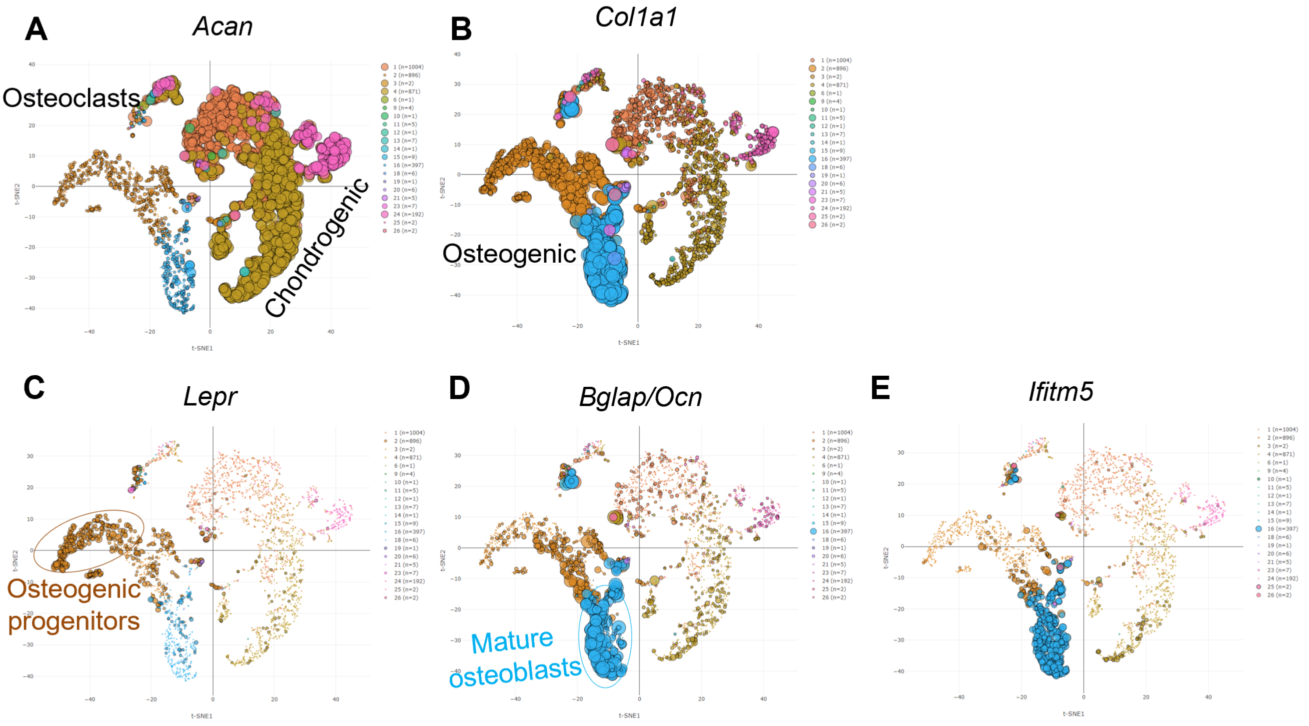

Figure S1. Expression profiles (tSNE) of osteogenic markers in single cell transcriptome generated from control mouse at P6 stage. Chondrogenic and osteogenic populations were identified. (A) *Acan* marks chondrogenic lineage. (B) *Col1a1* marks osteogenic lineage. (C) *Lepr* is expressed in osteogenic progenitors. (D) *Bglap* (Osteocalcin) is expressed in mature osteoblasts. (E) *Ifitm5* is expressed in mature osteoblasts.

Fig. S2

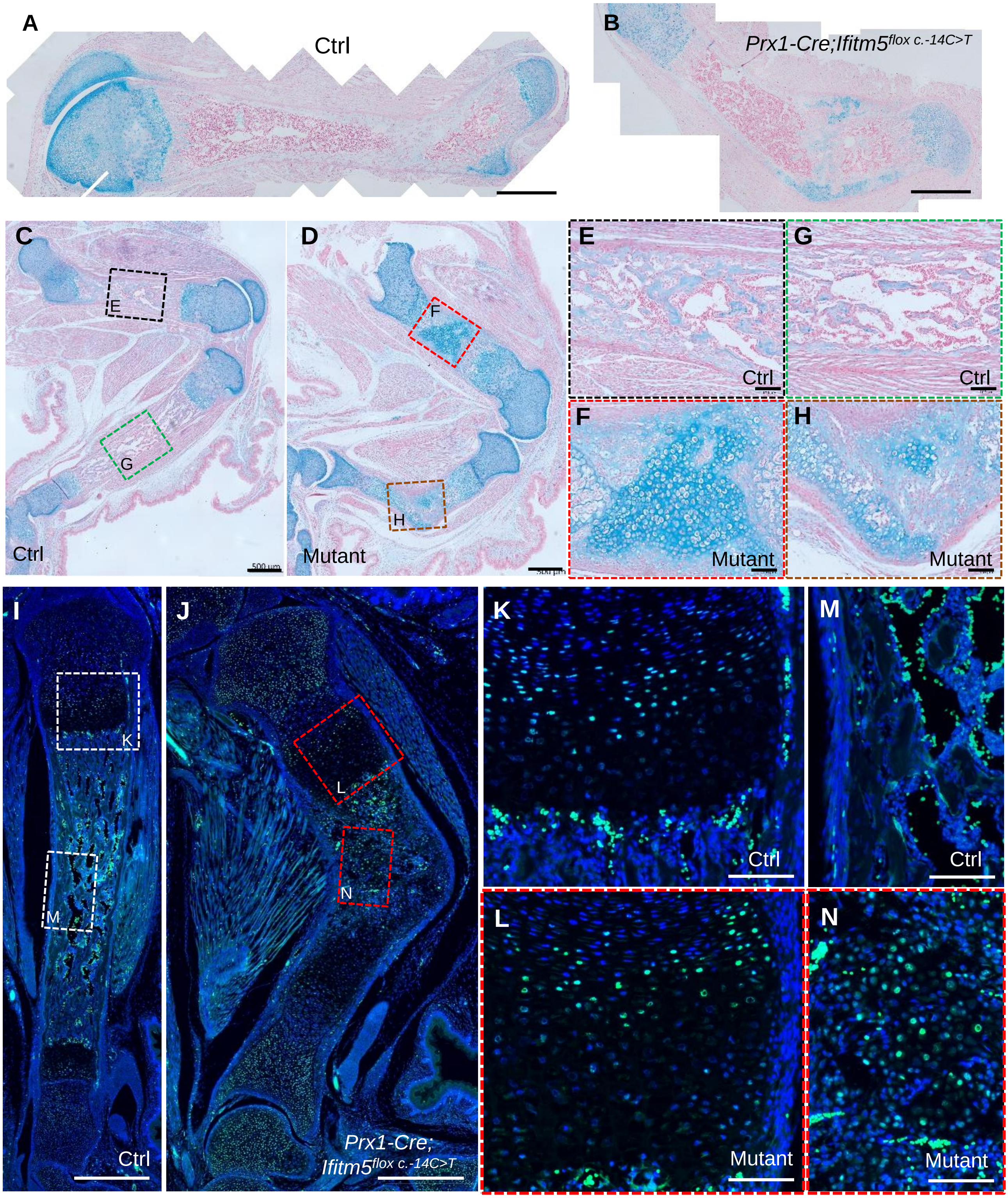

Figure S2. Aberrant chondrogenesis in *Prx1–Cre; Ifitm5*$^{flox\ c.-14C>T}$ mice. (A-B) Alcian blue staining of the femur sections from control and *Prx1–Cre; Ifitm5*$^{flox\ c.-14C>T}$ mice at P6 stage. (C-H) Alcian blue staining of femur and tibia sections from control and mutant mice at E18.5 stage. The diaphysis regions of the femur and tibia were shown at higher magnification. (I-N) Immuno-staining of SOX9 on the tibia sections from wild type and mutant mice at P1 stage. The boxed growth plate and cortical bone regions were shown at higher magnification. Scale bar in C-D and I-J = 500μm; Scale bar in A-B, E-H and K-N = 100μm.

Fig. S3

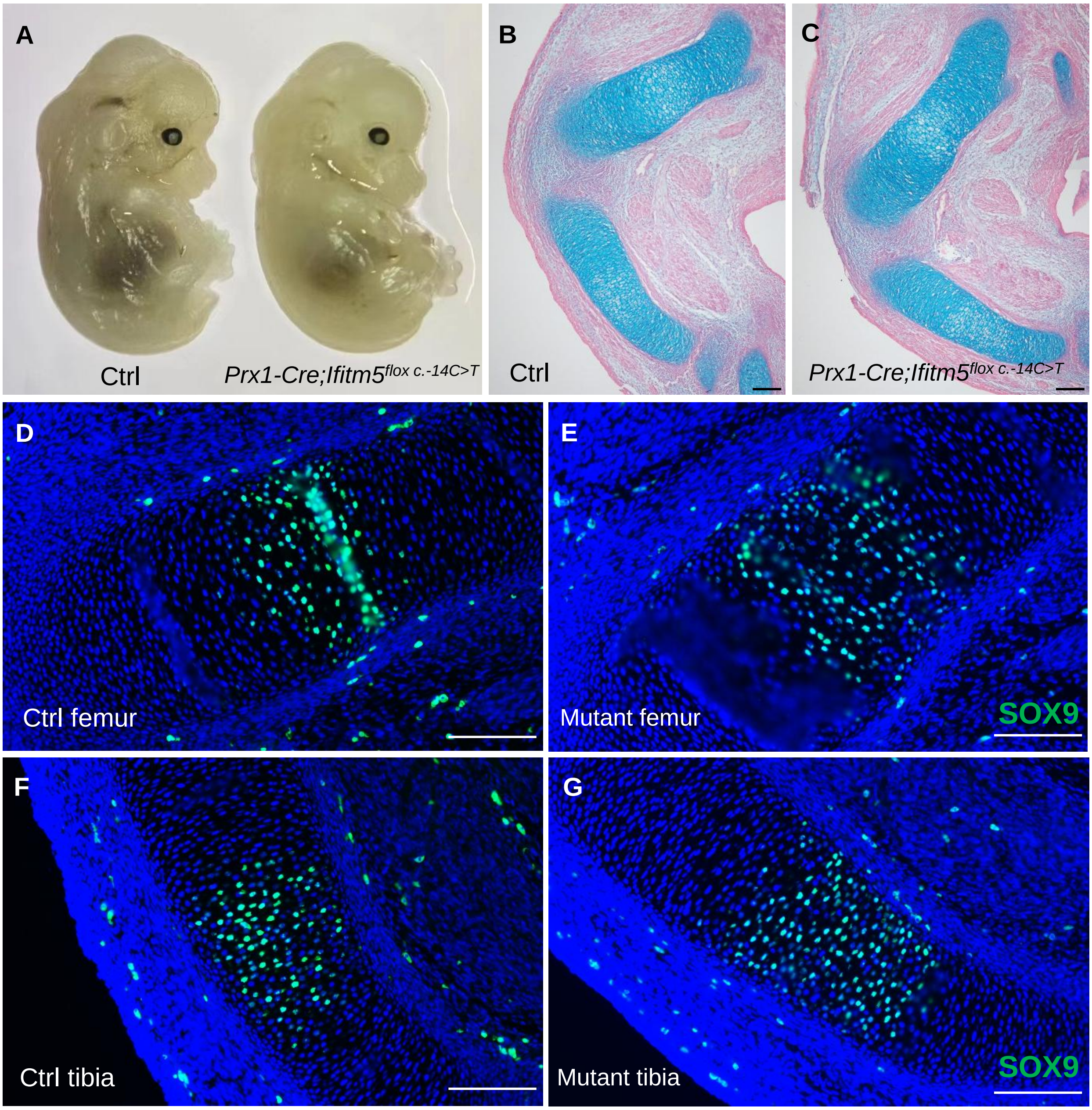

Figure S3 . Skeletal phenotypes of control and *Prx1–Cre; Ifitm5$^{flox\ c.\text{-}14C>T}$* mice. (A) Embryos of control and *Prx1–Cre; Ifitm5$^{flox\ c.\text{-}14C>T}$* mice at E14.5 stage from the same littermate. (B-C) Alcian blue staining of the hindlimbs from control and *Ifitm5$^{flox\ c.\text{-}14C>T}$* mice at E14.5 stage. (D-G) Immuno-staining of SOX9 on femur (D-E) and tibia (F-G) sections from control and mutant mice at E14.5 stage. Scale bar = 100μm.

Fig. S4

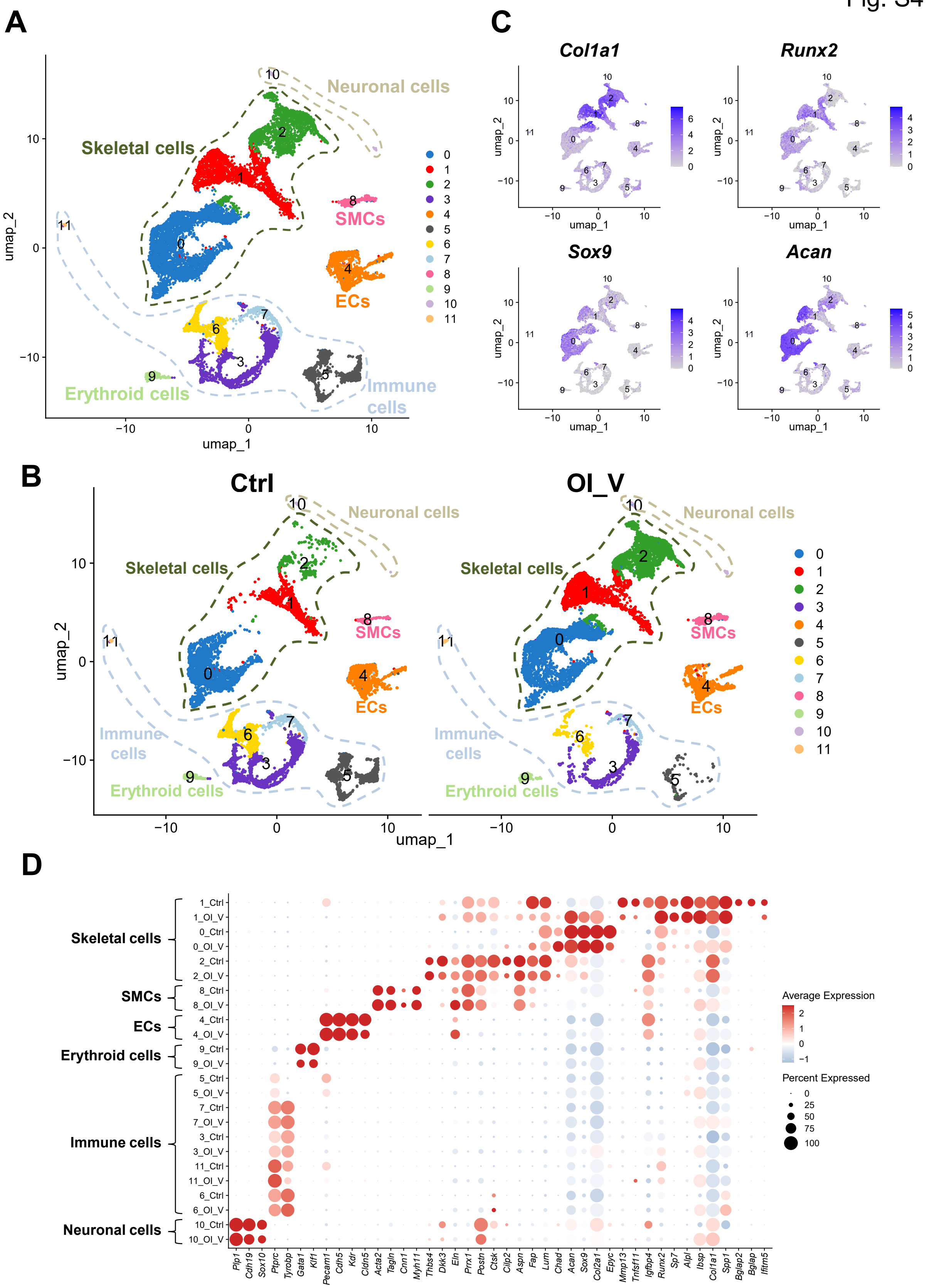
A
B
C
D
Neuronal cells
Skeletal cells
SMCs
ECs
Erythroid cells
Immune cells
umap_1
umap_2
Ctrl
OI_V
Col1a1
Runx2
Sox9
Acan
Average Expression
Percent Expressed
Plp1
Cdh19
Sox10
Ptprc
Tyrobp
Gata1
Klf1
Pecam1
Cdh5
Kdr
Cldn5
Acta2
Tagln
Cnn1
Myh11
Thbs4
Dkk3
Eln
Prrx1
Postn
Ctsk
Cilp2
Aspn
Fap
Lum
Chad
Acan
Sox9
Col2a1
Epyc
Mmp13
Tnfsf11
Igfbp4
Runx2
Sp7
Alpl
Ibsp
Col1a1
Spp1
Bglap2
Bglap
Ifitm5

Figure S4. Single-cell transcriptomic analyses of the tibiae from control (Ctrl) and *Prx1–Cre; Ifitm5*$^{flox\ c.\text{-}14C>T}$ mouse (OI_V) at P6 stage. (A-B) UMAP plots showing the annotated clusters of all the cells from the integrated (A) and separated samples (B). SMCs: smooth muscle cells; ECs: endothelial cells. (C) Feature plots showing the expression patterns of selected gene markers in the integrated UMAP. (D) Dot plot showing the expression patterns of selected cell-type specific markers in each cluster of each sample.

Fig. S5

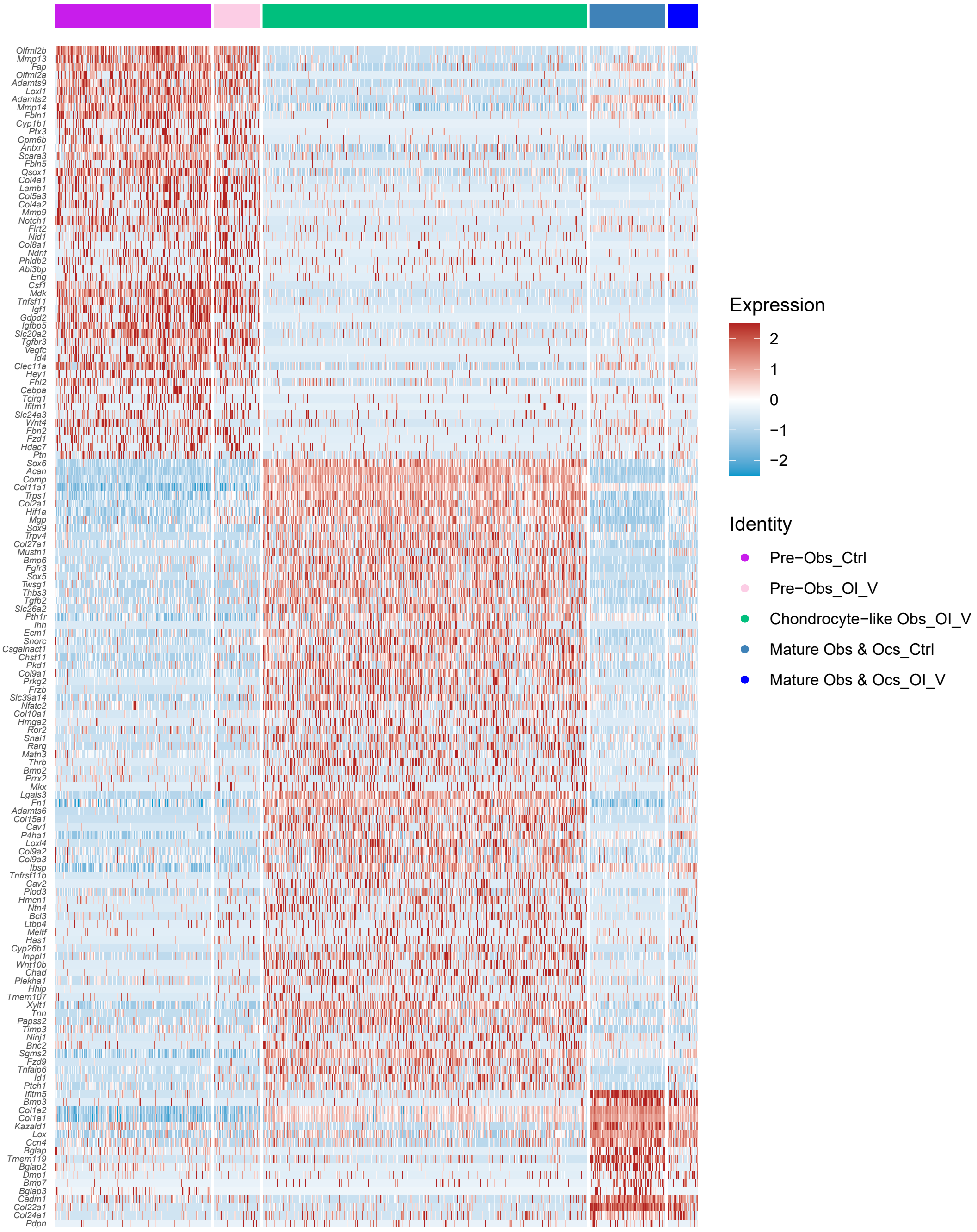

Figure S5. Heatmap showing the relative expression levels of the differentially expressed genes (DEGs) among the osteogenic populations (Pre-Obs, Chondrocyte-like Obs, Mature Obs & Ocs) in each sample. Obs: osteoblasts; Ocs: Osteocytes.

Fig. S6

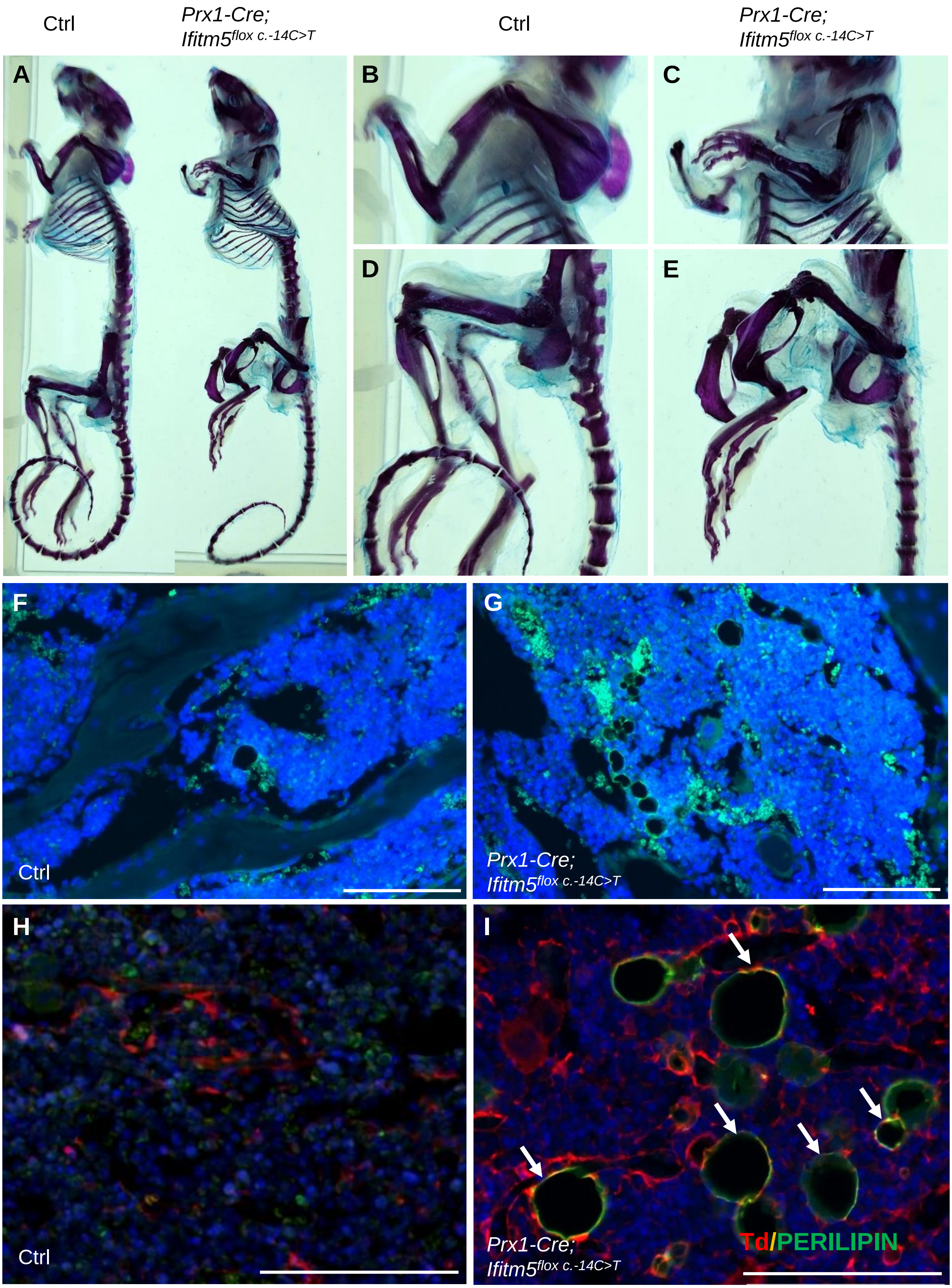
Ctrl
Prx1-Cre; Ifitm5flox c.-14C>T
Ctrl
Prx1-Cre; Ifitm5flox c.-14C>T
A
B
C
D
E
F
Ctrl
G
Prx1-Cre; Ifitm5flox c.-14C>T
H
Ctrl
I
Prx1-Cre; Ifitm5flox c.-14C>T
Td/PERILIPIN

Figure S6. Phenotypic analyses of adult *Prx1–Cre; Ifitm5$^{flox\ c.-14C>T}$* mice. (A) Skeletons of control and *Prx1–Cre; Ifitm5$^{flox\ c.-14C>T}$* mice stained with Alcian blue and Alizarin red at 8-week stage. (B-E) Regions of the forelimbs (B-C) and hindlimbs (D-E) were shown at higher magnification, respectively. (F-G) Immunostaining of PERILIPIN (Green) on the femur sections from control (F) and *Prx1–Cre; Ifitm5$^{flox\ c.-14C>T}$* (G) mice at 8-week stage. (H-I) Co-immunostaining of PERILIPIN (Green) and tdTomato (Red) on the distal tibia sections from control (H) and *Prx1–Cre; Ifitm5$^{flox\ c.-14C>T}$* (I) mice at 4-week stage. The adipocytes co-stained with PERILIPIN and Td were arrowed. Scale bar = 100µm.

Fig. S7

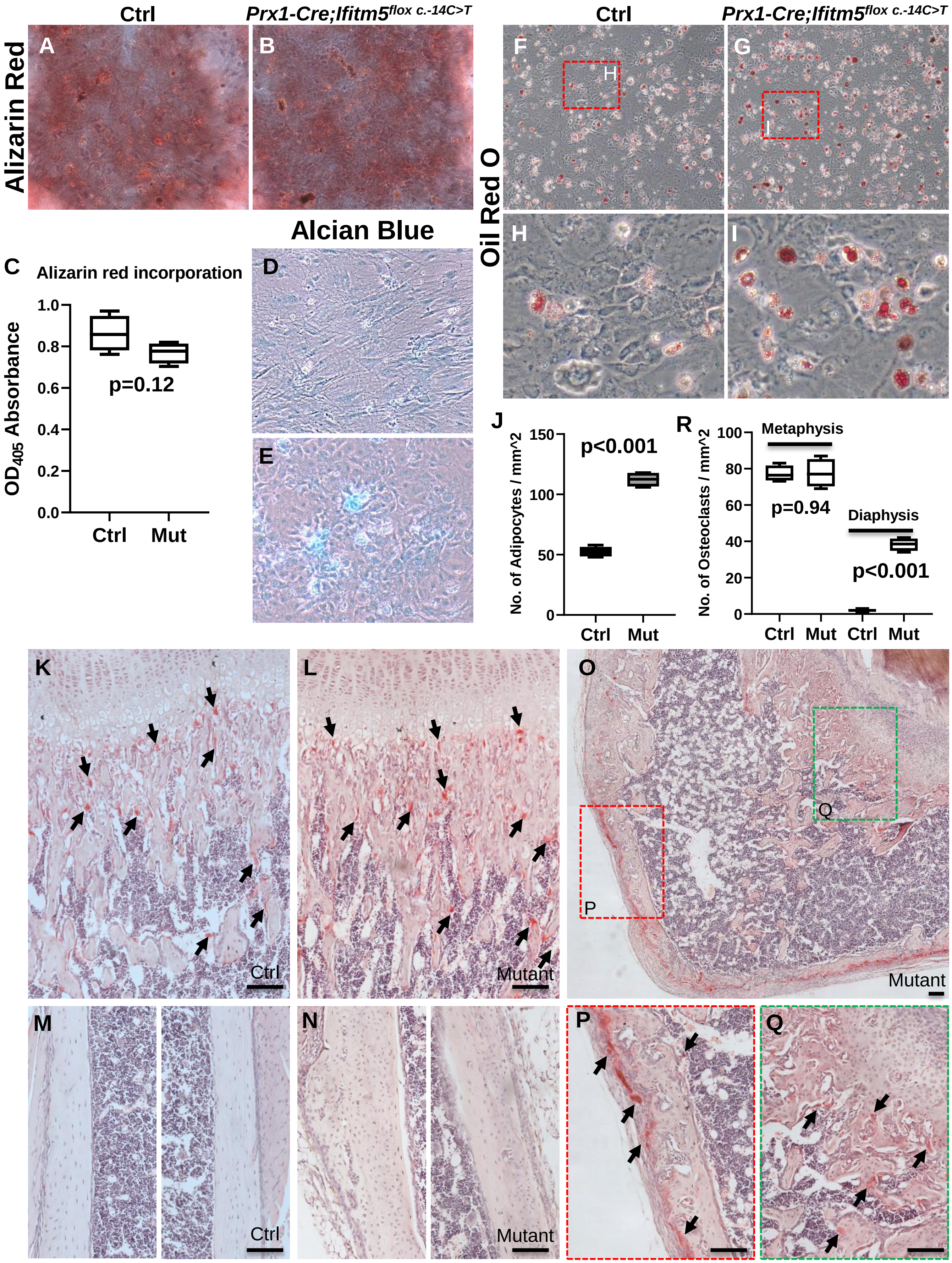

Ctrl
Prx1-Cre;Ifitm5flox c.-14C>T
Alizarin Red
A
B
C
Alizarin red incorporation
OD405 Absorbance
1.0
0.8
0.6
0.4
0.2
0.0
p=0.12
Ctrl
Mut
Alcian Blue
D
E
Oil Red O
F
G
H
I
J
No. of Adipocytes / mm^2
150
100
50
0
p<0.001
Ctrl
Mut
R
No. of Osteoclasts / mm^2
100
80
60
40
20
0
Metaphysis
p=0.94
Diaphysis
p<0.001
Ctrl
Mut
Ctrl
Mut
K
L
O
Q
P
Ctrl
Mutant
Mutant
M
N
P
Q
Ctrl
Mutant

Figure S7. *In vitro* differentiation of marrow mesenchymal stem cells and TRAP staining on the sections from control and *Prx1–Cre; Ifitm5$^{flox\ c.-14C>T}$* mice. (A-J) *In vitro* differentiation of mesenchymal stem cells isolated from the tibia marrow of control and *Prx1–Cre; Ifitm5$^{flox\ c.-14C>T}$* (Mut) mice at P6 stage. A-B: osteogenic differentiation (21-day), Alizarin red staining; C: Alizarin red staining rate was quantified by OD_{405} absorbance; D-E: chondrogenic differentiation (28-day), Alcian blue staining; F-I: adipogenic differentiation (28-day), Oil red O staining; J: Cells positive with oil red staining were quantified. (K-R) Tartrate resistant acid phosphatase (TRAP) staining on the tibia sections from control and *Prx1–Cre; Ifitm5$^{flox\ c.-14C>T}$* (Mut) mice at 4-week stage. K-L: trabecular regions; M-Q: cortical bone regions (TRAP-positive osteoclasts were arrowed. The boxed regions were shown at higher magnification); R: $TRAP^{+}$ osteoclasts were quantified. Scale bar = 100μm; Data were shown in boxplot; n = 4 for each sample; p-Values denoted statistical significance (Student's t-test, 2-tailed, unpaired).

Fig. S8

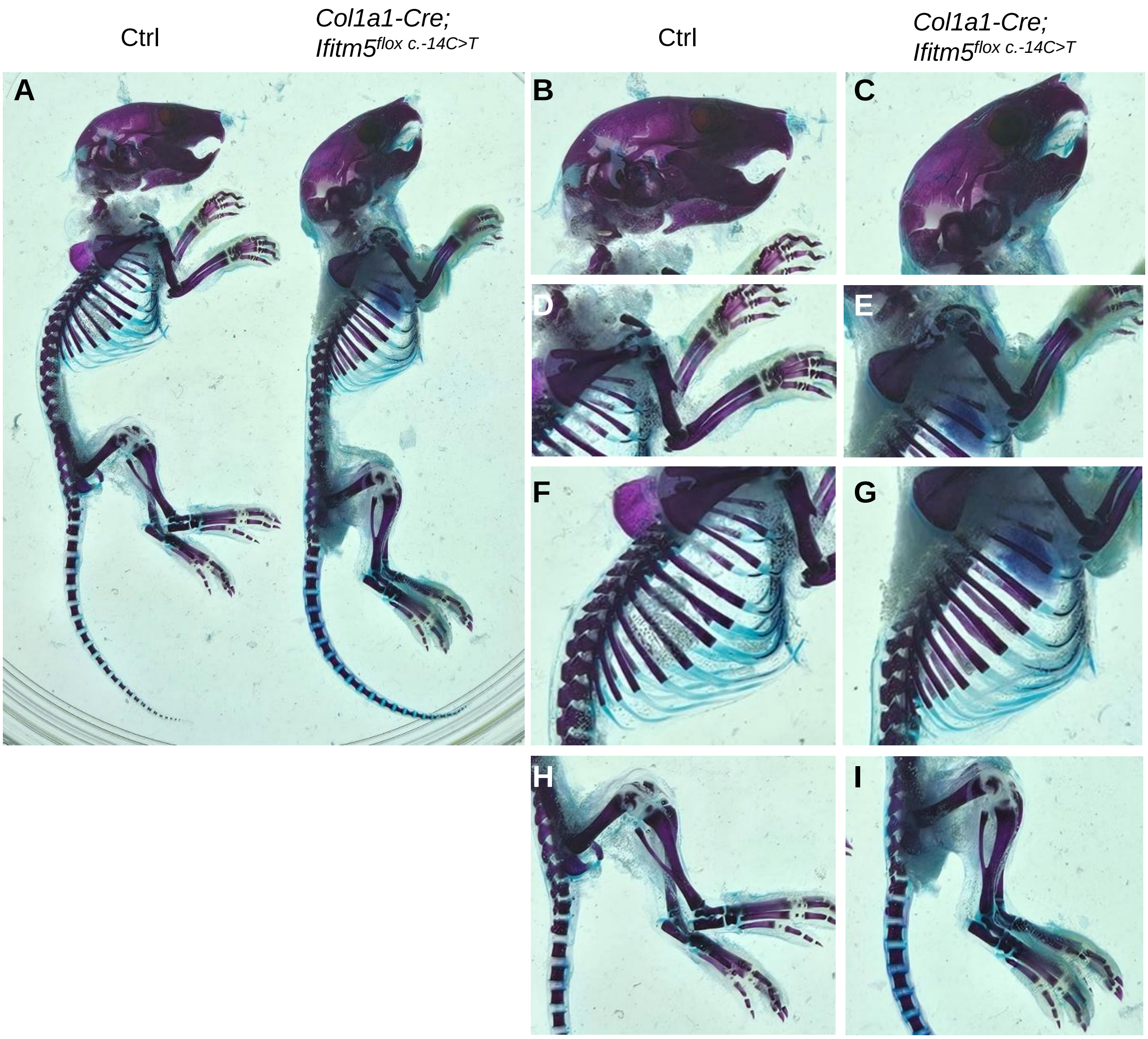

Figure S8. Skeletal morphology of control and *Col1a1–Cre; Ifitm5$^{flox\ c.-14C>T}$* mice. (A) Skeletons of control and *Col1a1–Cre; Ifitm5$^{flox\ c.-14C>T}$* mice stained with Alcian blue and Alizarin red at P10 stage. (B-I) Regions of the heads (B-C), forelimbs (D-E), ribcages (F-G) and hindlimbs (H-I) were shown at higher magnification, respectively.
