# Supplementary material for: An inducible mouse model of osteogenesis imperfecta type V reveals aberrant osteogenesis caused by Ifitm5 c.-14C>T mutation
Source: J Bone Miner Res. 2025 Feb 5;40(5):577–90. doi: 10.1093/jbmr/zjaf022 (PMC12103725; doi:10.1093/jbmr/zjaf022)
Supplement: Table_S1_Inducible_Ifitm5_Knock-in_design_zjaf022 [file table_s1_inducible_ifitm5_knock-in_design_zjaf022.docx]

| **Table S1. Wildtype *Ifitm5* genomic sequence** |
| --- |
| Red: exons; blue: gRNA; yellow: ATG; grey: c.-14C to be mutated. |
| 1 CAGGTA CGAATC TGAGGT CCCATT TATGCA TGTCTG CAGGGG TCATGG TCAAAG CCTGGA  61 GTCTGA GTTCTG GCAGGG ATATGC AGCTCT GATCCT GAGTGT GCCCTT CCCCCA GCCTTG  121 AGTAGG CTAAAC CGACCC ACAATG GTTTTA CAGCCT AGCCGT GGCCTC GCTCCA TTTGCA  181 ATTTCC TACAGG AGTGGA CTGCCC ACTGAG CTAGCT CTGCAA CATAAT CCAGCT GAAACA  241 AAGCAT GGGGTC TGTGGG CTTTCC CTCTGT AAACAC AGTGAT GAAAAT TAAACT TGGAGC  301 CTTGAT CAGAAA CTTTGT CTTGGC TTCATC CTTCTC TCTCAC CCCATT CCTTTT CATCTC  361 CAGCCC CCCTCT CAGGTC ACCCAG TTCACC CATGGC TGCTGG ACAGCC ACACCT GAGAGC  421 TCTGGT TGTTGC CTGAAA CTGATG CAGCCC CATGTT TTCTCA GCCCTA GTGTCT GCCATG  481 GAACTG TTTGCA GCCAGT GTTCTT GGGACA GCCTTT TGTGAA GTAGAG AGAGCA GCGGGA  541 CTCTGG TGGGTG GTCTAC AGCCAC TGCCCA TGTGGC GCAGGG GCCTTT GATGGG GTCTTT  601 TGAGAG GGGCGG GGGACG CTGGGG TGCAAG GGCGGG CCACTC TATTTG GCAGGC TGAGGC  661 TGTAAT TTGTGA TATATA TAGCGG GTGGAG CTGGTC AGCCAG GGAGGG TAGCAA GGGGCT  721 ATAAGC AGGCTT GGTGCT CAGAGA GGACAA GTCTCA GCTAGG AAGACA CGGCGC TGGAAC  781 CCATGG ACACTT CATATC CCCGTG AGGACC CCCGGG CTCCAT CATCCC GCAAGG CTGATG  841 CTGCAG CCCACA CAGCCC TCTCCA TGGGAA CACCTG GCCCTA CACCAC GAGATC ACATGC  901 TCTGGT CTGTCT TCAGCA CGATGT ACCTGA ATCTGT GCTGCC TTGGAT TCCTGG CGCTGG  961 TCCACT CTGTCA AGGTTG GCTTCA AGGCTT CTGGGA TGGGAG AATGAG TGTGGT GTGGGG  1021 GGAGGT GATGTG GAGTCC GGGAAG GAGGGG GCAGTG TTCAAA AGTATA TGAGGG TGTCTG  1081 TTGGCA TAGCCA CAGTAC CTCACC AAGTTT GGGGAC CGTTAA GGGGTG TATGCA CTGTTC  1141 ACAGGA AAGGAA GGTCTG GCTATA TAGTCT CCCTCA AAGGGC AAAGTC CTTAAA GGTTGC  1201 CCATAG AGGAAT TCCCAC TGTAGT TCCCGA ATAGCA CTGAGT TCTTGG TTTCTC ATAGGA  1261 CTCCTC TCCCCT AGGAGG CTCTCA GAGGGT GCTGCT GACCCA GAATGG CCACTG AATCTC  1321 TACCTG CAGCCT TTATTT ATGCTG AGACTA TGGTTA GGACCC TGCCAG AGGCCA AACTGT  1381 GCCTGT GAAGGA ACTAGG TCCAGC CGAGGG ATATGT CTCCTT GAGCCA GAGTCC TCCGTC  1441 AAGATT TAGAGT TTAGCC TGTGCT CTACAG TTGCTC CGGGGG AGGGGG GGTCCC CTAGAC  1501 ACCATG AACATG TTAACA TCTTGT TTCTGG TGCTGC CCTGCA GGCCCG AGACCA GAAGAT  1561 GGCTGG GAACTT GGAGGC TGCAAG GCAGTA TGGCTC CAAAGC CAAGTG CTACAA CATCCT  1621 GGCTGC AATGTG GACATT GGTGCC CCCATT GCTGCT CCTGGG ACTGGT GGTGAC TGGCGC  1681 CTTGCA CCTGTC CAAGTT AGCCAA AGACTC TGCGGC TTTCTT CAGCAC CAAGTT TGATGA  1741 GGAGGA CTATAA CTAAGA GTTCCG AGCCTG TCCCTG AACCGA GGACAA CCATGT CAGGTC  1801 AGCTGT GCCCAA CACCAG CTCCTG GGAGTT ACAGCC TACTAT AGCACC TCGATC CCTGCC  1861 CAGCCG TGACCT CAGAGG CGGCCC TCTTGT CCACGA TGTGTA TTTGAT GCTGCC CCACTC  1921 AGATCC CTCAGA CTTAAC TTATAA CCTAGA GGGTCC TCAGTG CTTGGC TTTGTC CCTTTC  1981 CCCACT TACCAG GCCGAG CCTCTG TTCACG ATTAAA AGTGGC AGATTC CAGAAA TGTGTG  2041 GGCTCT TCCTTT CCTGGT GGTAGT TGTGTA GGGAGC CCCAGC ACCAAC TCATCT GGCCAA  2101 CCCCAG TTATCT TATTCT GTTAGC TTGGCT GTCCGA CTACTG CTTTTG CTTAGC GGCCTG  2161 TGGCTT CTGGGG TGGAGG AGCCTG AGTATT TTAGGA TGAAGT TCGGGA ACGCAG GCCTTT  2221 GATTCC CACTGC CCTGTG GTTTGA TGGGCT GTGTCC GAGAAC ATGTGA GGGTGT GCCCTG  2281 ACTCCT CATCCG CCACAG TGGTCT TCTTGG ACTGTG CGGAAG GCAGAC AGGGGC CATTTC |

| **Table S1. *Ifitm5* Cas9-KI targeted vector sequence** |
| --- |
| **Red:** Knock-in fragment; underlined: LoxP sites; 683~691bp, 3041~ 3049bp: *Kozak* sequence; EGFP: 692~1411bp; 3*stop: 1412~3005bp. |

1 AGTAGG CTAAAC CGACCC ACAATG GTTTTA CAGCCT AGCCGT GGCCTC GCTCCA TTTGCA

61 ATTTCC TACAGG AGTGGA CTGCCC ACTGAG CTAGCT CTGCAA CATAAT CCAGCT GAAACA

121 AAGCAT GGGGTC TGTGGG CTTTCC CTCTGT AAACAC AGTGAT GAAAAT TAAACT TGGAGC

181 CTTGAT CAGAAA CTTTGT CTTGGC TTCATC CTTCTC TCTCAC CCCATT CCTTTT CATCTC

241 CAGCCC CCCTCT CAGGTC ACCCAG TTCACC CATGGC TGCTGG ACAGCC ACACCT GAGAGC

301 TCTGGT TGTTGC CTGAAA CTGATG CAGCCC CATGTT TTCTCA GCCCTA GTGTCT GCCATG

361 GAACTG TTTGCA GCCAGT GTTCTT GGGACA GCCTTT TGTGAA GTAGAG AGAGCA GCGGGA

421 CTCTGG TGGGTG GTCTAC AGCCAC TGCCCA TGTGGC GCAGGG GCCTTT GATGGG GTCTTT

481 TGAGAG GGGCGG GGGACG CTGGGG TGCAAG GGCGGG CCACTC TATTTG GCAGGC TGAGGC

541 TGTAAT TTGTGA TATATA TAGCGG GTGGAG CTGGTC AGCCAG GGAGGG TAGCAA GGGGCT

601 ATAAGC AGGCTT GGTGCT CAGAGA GGACAA GTCTCA GCTAGG AAGACA TAACTT CGTATA

661 GCATAC ATTATA CGAAGT TATAGC CGCCAC CATGGT GAGCAA GGGCGA GGAGCT GTTCAC

721 CGGGGT GGTGCC CATCCT GGTCGA GCTGGA CGGCGA CGTAAA CGGCCA CAAGTT CAGCGT

781 GTCCGG CGAGGG CGAGGG CGATGC CACCTA CGGCAA GCTGAC CCTGAA GTTCAT CTGCAC

841 CACCGG CAAGCT GCCCGT GCCCTG GCCCAC CCTCGT GACCAC CCTGAC CTACGG CGTGCA

901 GTGCTT CAGCCG CTACCC CGACCA CATGAA GCAGCA CGACTT CTTCAA GTCCGC CATGCC

961 CGAAGG CTACGT CCAGGA GCGCAC CATCTT CTTCAA GGACGA CGGCAA CTACAA GACCCG

1021 CGCCGA GGTGAA GTTCGA GGGCGA CACCCT GGTGAA CCGCAT CGAGCT GAAGGG CATCGA

1081 CTTCAA GGAGGA CGGCAA CATCCT GGGGCA CAAGCT GGAGTA CAACTA CAACAG CCACAA

1141 CGTCTA TATCAT GGCCGA CAAGCA GAAGAA CGGCAT CAAGGT GAACTT CAAGAT CCGCCA

1201 CAACAT CGAGGA CGGCAG CGTGCA GCTCGC CGACCA CTACCA GCAGAA CACCCC CATCGG

1261 CGACGG CCCCGT GCTGCT GCCCGA CAACCA CTACCT GAGCAC CCAGTC CGCCCT GAGCAA

1321 AGACCC CAACGA GAAGCG CGATCA CATGGT CCTGCT GGAGTT CGTGAC CGCCGC CGGGAT

1381 CACTCT CGGCAT GGACGA GCTGTA CAAGTA ACTGTA AGTCTG CAGAAA TTGATG ATCTAT

1441 TAAACA ATAAAG ATGTCC ACTAAA ATGGAA GTTTTT CCTGTC ATACTT TGTTAA GAAGGG

1501 TGAGAA CAGAGT ACCTAC ATTTTG AATGGA AGGATT GGAGCT ACGGGG GTGGGG GTGGGG

1561 TGGGAT TAGATA AATGCC TGCTCT TTACTG AAGGCT CTTTAC TATTGC TTTATG ATAATG

1621 TTTCAT AGTTGG ATATCA TAATTT AAACAA GCAAAA CCAAAT TAAGGG CCAGCT CATTCC

1681 TCCCAC TCATGA TCTATA GATCTA TAGATC TCTCGT GGGATC ATTGTT TTTCTC TTGATT

1741 CCCACT TTGTGG TTCTAA GTACTG TGGTTT CCAAAT GTGTCA GTTTCA TAGCCT GAAGAA

1801 CGAGAT CAGCAG CCTCTG TTCCAC ATACAC TTCATT CTCAGT ATTGTT TTGCCA AGTTCT

1861 AATTCC ATCAGA AGCTTG CAGATC TGCGAC TCTAGA GGATCG ACTGTG CCTTCT AGTTGC

1921 CAGCCA TCTGTT GTTTGC CCCTCC CCCGTG CCTTCC TTGACC CTGGAA GGTGCC ACTCCC

1981 ACTGTC CTTTCC TAATAA AATGAG GAAATT GCATCG CATTGT CTGAGT AGGTGT CATTCT

2041 ATTCTG GGGGGT GGGGTG GGGCAG GACAGC AAGGGG GAGGAT TGGGAA GACAAT AGCAGG

2101 CATGCT GGGGAT GCGGTG GGCTCT ATGGCT GCGACT CTAGAG GATCAT AATCAG CCATAC

2161 CACATT TGTAGA GGTTTT ACTTGC TTTAAA AAACGT TTAAAC CTCCCA CACCTC CCCCTG

2221 AACCTG AAACAT AAAATG AATGCA ATTGTT GTTGTT AACTTG TTTATT GCAGCT TATAAT

2281 GGTTAC AAATAA AGCAAT AGCATC ACAAAT TTCACA AATAAA GCATTT TTTTCA CTGCAT

2341 TCTAGT TGTGGT TTGTCC AAACTC ATCAAT GTATCT TATCAT GTCTGG ATCTGC GACTCT

2401 AGAGGA TCATAA TCAGCC ATACCA CATTTG TAGAGG TTTTAC TTGCTT TAAAAA ACCTCC

2461 CACACC TCCCCC TGAACC TGAAAC ATAAAA TGAATG CAATTG TTGTTG TTAACT TGTTTA

2521 TTGCAG CTTATA ATGGTT ACAAAT AAAGCA ATAGCA TCACAA ATTTCA CAAATA AAGCAT

2581 TTTTTT CACTGC ATTCTA GTTGTG GTTTGT CCAAAC TCATCA ATGTAT CTTATC ATGTCT

2641 GGATCT GCGACT CTAGAG GATCAT AATCAG CCATAC CACATT TGTAGA GGTTTT ACTTGC

2701 TTTAAA AAACCT CCCACA CCTCCC CCTGAA CCTGAA ACATAA AATGAA TGCAAT TGTTGT

2761 TGTTAA CTTGTT TATTGC AGCTTA TAATGG TTACAA ATAAAG CAATAG CATCAC AAATTT

2821 CACAAA TAAAGC ATTTTT TTCACT GCATTC TAGTTG TGGTTT GTCCAA ACTCAT CAATGT

2881 ATCTTA TCATGT CTGGAT CCCCAT CAAGCT GATAAC ATACGC TCTCCA TCAAAA CAAAAC

2941 GAAACA AAACAA ACTAGC AAAATA GGCTGT CCCCAG TGCAAG TGCAGG TGCCAG AACATT

3001 TCTCTA TAACTT CGTATA GCATAC ATTATA CGAAGT TATCGC CGCCAC CATGGC GCTGGA

3061 ACCCAT GGACAC TTCATA TCCCCG TGAGGA CCCCCG GGCTCC ATCATC CCGCAA GGCTGA

3121 TGCTGC AGCCCA CACAGC CCTCTC CATGGG AACACC TGGCCC TACACC ACGAGA TCACAT

3181 GCTCTG GTCTGT CTTCAG CACGAT GTACCT GAATCT GTGCTG CCTTGG ATTCCT GGCGCT

3241 GGTCCA CTCTGT CAAGGT TGGCTT CAAGGC TTCTGG GATGGG AGAATG AGTGTG GTGTGG

3301 GGGGAG GTGATG TGGAGT CCGGGA AGGAGG GGGCAG TGTTCA AAAGTA TATGAG GGTGTC

3361 TGTTGG CATAGC CACAGT ACCTCA CCAAGT TTGGGG ACCGTT AAGGGG TGTATG CACTGT

3421 TCACAG GAAAGG AAGGTC TGGCTA TATAGT CTCCCT CAAAGG GCAAAG TCCTTA AAGGTT

|  |
| --- |
